# Rapid Renin Angiotensin Aldosterone System inhibitor optimisation in patients with chronic kidney disease and heart failure with reduced ejection fraction using potassium binder: a randomised, double-blinded, placebo-controlled trial

**DOI:** 10.1038/s41467-026-78377-0

**Published:** 2026-10-14

**Authors:** Debasish Banerjee, Mahrukh Ali, Ella Tumelty, Daniel Murphy, Hilary Warrens, Simran Parmar, Thomas McNally, Tony Lopez, Sharirose Abat, Riny Paul, Sabba Hussain, Souradip Mookerjee, Isaac Chung, Irina Chis Ster, Lisa Anderson

**Affiliations:** 1 Renal and Transplantation Unit, St George’s University Hospitals NHS Foundation Trust, London, UK; 2 Department of Medicine, School of Health and Medical Sciences, City St George’s University of London, London, UK

**Keywords:** Cardiology, Nephrology

## Abstract

Renin-angiotensin-aldosterone inhibitors (RAASi) improve outcomes in patients with heart failure with reduced ejection fraction (HFrEF), but their use is often limited by hyperkalaemia in chronic kidney disease (CKD) patients. This double-blind, placebo-controlled, randomised trial evaluated whether sodium zirconium cyclosilicate (SZC) enables RAASi up-titration in HFrEF patients with CKD stages 3–5 and serum potassium of 5.0–5.5 mmol/L over 14 weeks. The primary binary outcome was defined as achieving the target dose of RAAS inhibition therapy without reaching hyperkalaemia (serum potassium ≥5.6 mmol/L) over 14 weeks or otherwise. Participants (*n* = 112) were randomised 1:1 to receive sodium zirconium cyclosilicate (SZC) or placebo. The per-protocol (PP) analyses included 43 out of 53 (81%) in the SZC arm and 53 out of 59 participants (90%) in the placebo arm; the intention-to-treat (ITT) analyses included all 112. At the end of the trial, 24 of 43 participants (56%) in the SZC group achieved the target RAASi dose without developing hyperkalaemia compared with 15 of 53 patients (28%) in the placebo group (PP analysis, *p* = 0.006, ITT analysis, *p* = 0.007,). Participants in the SZC group were 3 times more likely to reach the target dose without reaching potassium 5.6 mmol/L (OR = 3.16; 95% CI 1.35–7.39; *p* = 0.008) than in the placebo group (ITT analysis). No significant differences in adverse events between the two groups were observed. SZC appears to safely support rapid RAASi optimisation in HFrEF patients with CKD. This trial was funded by AstraZeneca via Externally Sponsored Scientific Research grant ESR-19-20174. Trial Registration: Clinicaltrials.gov NCT05004363 and EudraCT number 2020-002946-18.

## Abstract (web summary)

In this randomized controlled trial, authors show that sodium zirconium cyclosilicate helped people with heart failure and chronic kidney disease reach recommended doses of key heart failure medicines, while reducing episodes of high blood potassium without added safety concerns.

## Introduction

Renin angiotensin aldosterone system inhibitors (RAASi) including mineralocorticoid receptor antagonist (MRA) with either angiotensin-converting-enzyme inhibitor (ACEi), angiotensin-receptor blocker (ARB), or angiotensin-receptor-neprilysin inhibitor (ARNi), are essential guideline-directed medical therapy to improve outcomes in heart failure patients with reduced ejection fraction (HFrEF)^1^. Maximisation of such therapy is recommended and requires close monitoring of symptoms, signs and laboratory parameters^2^.

Chronic kidney disease (CKD) affects approximately half of the patients with heart failure and is associated with worse outcomes^3^. CKD-associated hyperkalaemia and changes in eGFR due to RAASi limit their use in heart failure patients, who will benefit from RAASi the most^4^. Hyperkalaemia in CKD patients increases with heart failure, diabetes, advanced stages of kidney disease and RAASi use; and is associated with higher hospitalisations, increased healthcare costs, and RAASi discontinuation. RAASi discontinuation, in turn, was found to be associated with higher mortality^5^.

The new potassium binders, including Sodium Zirconium Cyclosilicate (SZC), enable RAASi maximisation in HFrEF with modest reduction of potassium levels^6^. SZC has been shown to reduce serum potassium levels and maintain lower levels^7,8^. However, SZC has not been tested in advanced CKD patients with heart failure for RAASi maximisation.

In a recently completed study utilising SZC in HFrEF patients, a higher proportion of patients achieved a target dose of spironolactone with normokalaemia >25 mg; however, the study excluded patients with eGFR<30 ml/min/1.73m2^9^.

The present study investigated the role of SZC in RAASi maximisation in HFrEF patients with chronic kidney disease, including patients with eGFR<30 ml/min/1.73 m^2^ and serum potassium of 5.0 to 5.5 mmol/L^10^.

## Results

**Fig. 1:**
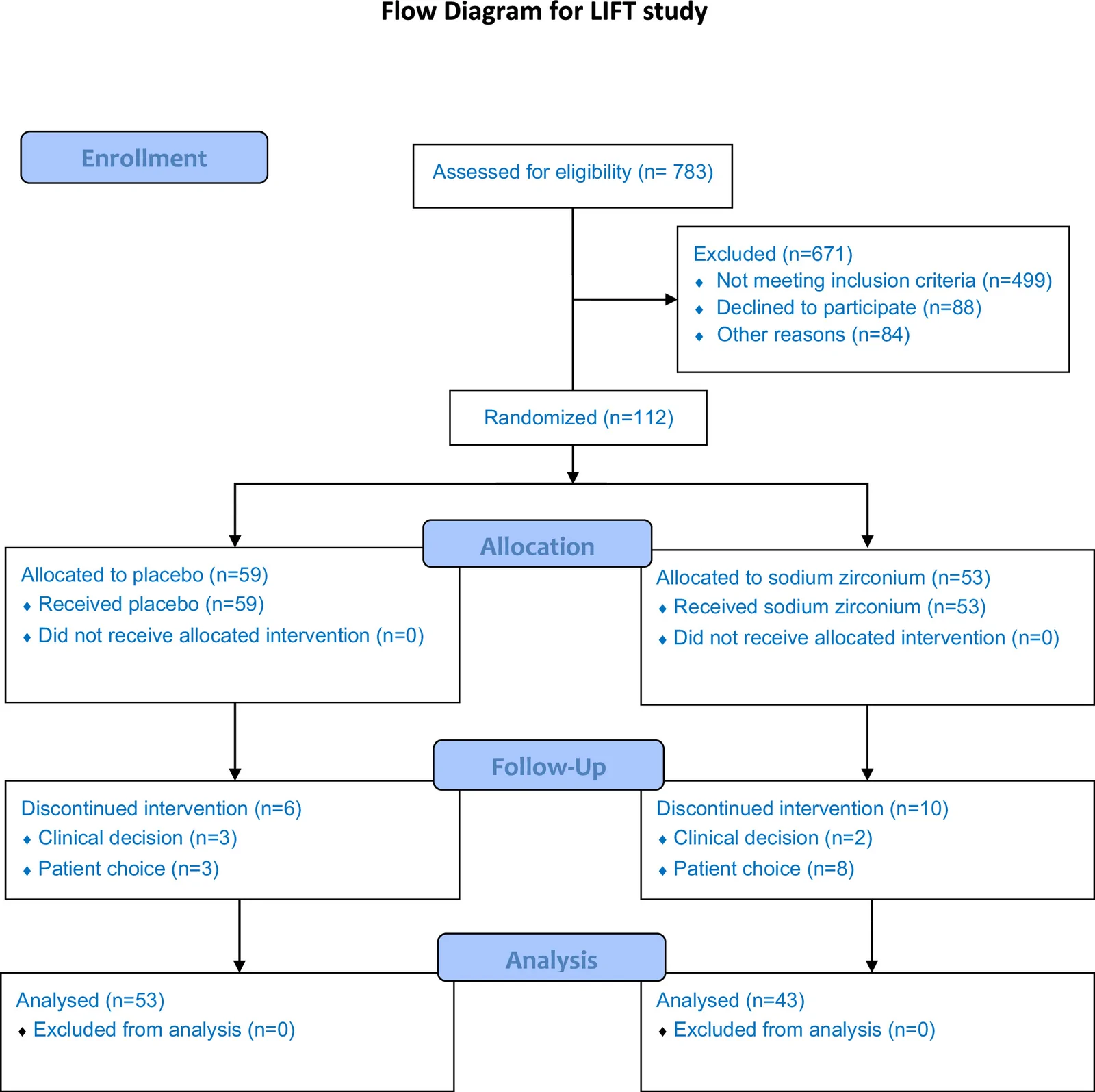
Consort Diagram Showing enrolment, random allocation and follow-up of participants in the study.

112 patients were randomised to SZC (*n* = 53) or placebo (*n* = 59) with age 73±11years, 27% females; 54% diabetics, 54% hypertensive, 62% with IHD history, 28% obese, and 53% with hypercholesterolaemia (Fig. 1). Overall baseline laboratory markers included potassium 4.98 ± 0.38 mmol/L, eGFR 35 ± 12 ml/min/1.73m^2^, Troponin 31 (IQR 21 – 52), ng/L, NT-pro BNP 1683 (IQR 720, 4102) pg/L and (log scale) albumin-creatinine-ratio (ACR) 1.73 ± 1.51 mg/mmol. Table 1 presents these summaries stratified by the two intervention groups as well as for Troponin, NT-pro BNP and ACR (Full table in Supplementary Table S1). Six out of 59 patients (10%) in the placebo group and 10 out of 53 (19%) patients in the SZC group withdrew from the study, and there was no significant difference between the distribution of the withdrawals between the two groups (*p* = 0.189).

**Table 1:** Baseline characteristics at randomisation

Baseline Characteristics	Summaries/Categories	Placebo (*n* = 59)	Sodium Zirconium (*n *= 53)
DEMOGRAPHICS			
Age (years)	Median (Q1-Q3)	73(66-80)	76(69-81)
Gender	Male	44(75%)	38(72%)
Ethnicity	White	41 (69%)	34 (64%)
ASSOCIATED CHRONIC CONDITIONS			
Diabetes	Yes	29 (49%)	32 (60%)
Hypertension	Yes	35 (59%)	25 (47%)
IHD history	Yes	34 (58%)	35 (66%)
	Missing	0 (0%)	1 (2%)
Smoker	Ever	34 (58%)	36 (68%)
Obesity	Yes	15 (25%)	16 (30%)
Hypercholesterolemia	Yes	32 (54%)	27 (51%)
	Missing	0(0%)	2 (4%)
CKD Stage	CKD 3a	15 (25%)	10 (18.9%)
	CKD 3b	22 (37%)	26 (49.1%)
	CKD 4	22 (37%)	15 (28.3%)
	CKD 5	0 (0%)	2 (3.8%)
LABORATORY MARKERS			
Sodium	Mean/SD	138.97/2.99	138.75/3.29
Potassium levels (baseline)	Mean/SD	4.93/0.36	5.03/0.39
eGFR (baseline)	Median (Q1-Q3)	34(27- 45)	33(28- 41)
Troponin	Median (Q1-Q3)	29.5(22, 45)	35(19.5, 55.5)
	Min-Max	9-582	7-227
	Missing	1 (1.7%)	1(1.9%)
NTproBNP scale	Median (Q1-Q3)	1683(665, 3686)	1685.5(797, 6234)
	Min-Max	95-32594	89-31706
	Missing	1 (1.7%)	1(1.9%)
Urine albumin creatinine ratio (original scale)	Median (Q1-Q3)	3.35(1.8, 12.9)	4.4(2.7, 16.6)
	Min-Max	0.4-514.3	0.8-388.7
	Missing	11(18.6%)	12(22.6%)
GUIDELINE-BASED MEDICAL THERAPY PRIOR TO RANDOMISATION			
Betablockers	Yes, any dose	53 (89.8%)	51 (96.2%)
ACEi/ARB/ARNI	Yes, any dose	48 (81.4%)	41 (77.4%)
MRA	Yes, any dose	27 (45.8%)	20 (37.7%)
SGLT2i	Yes, any dose	37 (62.7%)	38 (71.7%)

Baseline characteristics at randomisation, including laboratory measurements. Categorical and binary variables are summarised as numbers and percentages; continuous variables as means, standard deviations, medians, and interquartile ranges. Statistics are stratified by intervention group.

*NTproBNP* N-terminal pro-brain natriuretic peptide, *ACEi/ARB/ARNI* angiotensin-converting-enzyme inhibitor/angiotensin-receptor blocker/angiotensin-receptor-neprilysin inhibitor, *MRA* mineralocorticoid receptor antagonist, *SGLT2i* sodium-glucose transporter 2 inhibitor.

### Primary outcome

**Fig. 2:**
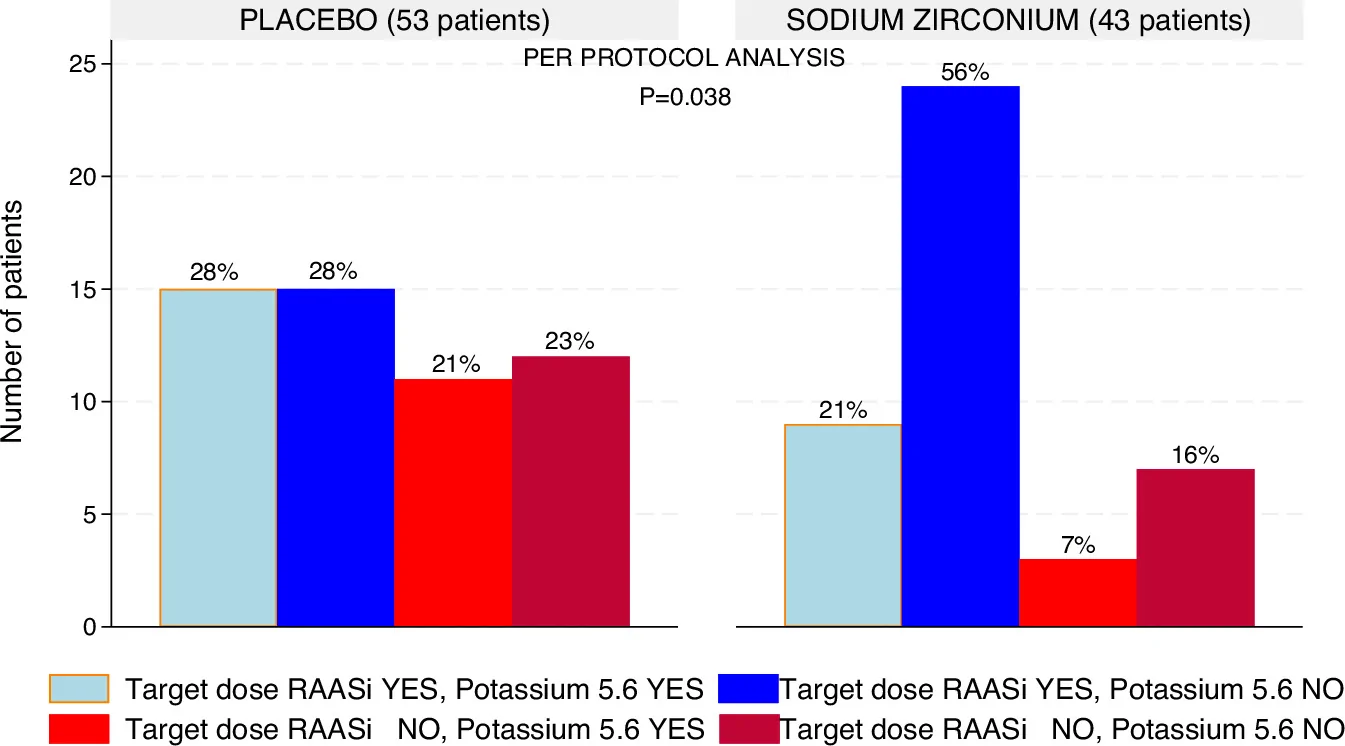
Distribution of the primary outcome by intervention group. The binary primary outcome—defined as achieving the target dose of RAASi while maintaining potassium levels below 5.6 mmol/L versus any other situation—was further refined into four categories based on the cross-classification of its two components: achieving the target RAASi dose (yes/no) and maintaining potassium levels below 5.6 mmol/L (yes/no). The plot reflects the per-protocol analysis. The proportion of responders in each group is illustrated by the dark blue bars. Chi-squared tests for binary/categorical outcomes for independent groups were used. The p-values and the sample sizes are displayed in Table 2, including those corresponding to ITT analyses. PP analysis for the main binary outcome: χ^2^ = 7.45, DF = 1, p = 0.006, *n* = 96.; ITT analysis *p* = 0.007, *n* = 112. This is a randomisation/permutation test (based on 5000 simulations).PP analysis for the main detailed categorical outcome: χ^2^ = 8.51, DF = 3, p = 0.038, n = 96.

At the end of the trial, 24 of 43 patients (56%) in the SZC group achieved the target RAASi dose without developing hyperkalaemia (Table 2), compared with 15 of 53 patients (28%) in the placebo group (intention-to-treat analysis [ITT] analysis, *p* = 0.007, per-protocol analysis, *p* = 0.006). Patients in the SZC group were more than 3 times more likely to reach the target dose without reaching potassium 5. 6 mmol/L (OR = 3.16; 95%CI 1.35–7.39; *P* = 0.008) than in the placebo group ITT analysis. A refined per-protocol analysis of the composite outcome (Fig. 2, Table 2) similarly demonstrated a significant difference between the two groups (*P* = 0.038), corroborated by the corresponding ITT analysis (*P* = 0.039). Supplementary Table 2 presents sensitivity analyses for the trial’s primary outcome.

**Table 2:** Per-protocol (PP, N = 96) and intention-to-treat (ITT, N = 112) analyses were conducted for the primary composite endpoint—achieving the target RAASi dose while maintaining serum potassium below 5.6 mmol/L—and its refined four-category version, which classifies participants by whether they reached the target dose and whether they maintained potassium below 5.6 mmol/L

Outcome		PER PROTOCOL ANALYSIS (96 PATICIPANTS)					ITT (*N* = 112)
		Placebo (*N* = 53)		Intervention (*N *= 43)		*P*- value	*P*-value
Variables	Category	Summary		Summary			
		Number	%	Number	%		
PRIMARY OUTCOME (binary)	Reached target dose =YES Reached Potassium 5.6 = NO	15	(28%)	24	(56%)	0.006	0.007
DF = = 1	All other possibilities	38	(72%)	19	(44%)		
PRIMARY OUTCOME (refined categorical)	Reached target dose =YES Reached Potassium 5.6 = YES	15	(28%)	9	(21%)	0.038	0.039
DF = = 3	Reached target dose =YES Reached Potassium 5.6 = NO	15	(28%)	24	(56%)		
	Reached target dose =NO Reached Potassium 5.6 = YES	11	(21%)	3	(7%)		
	Reached target dose =NO Reached Potassium 5.6 = NO	12	(23%)	7	(16%)		
SECONDARY OUTCOMES							
DF = = 1	Reached target dose =YES Reached Potassium 6 = NO	24	(45%)	30	(70%)	0.016	0.035
	All other possibilities	29	(55%)	13	(30%)		
SECONDARY OUTCOME (refined categorical)	Reached target dose =YES Reached Potassium 6 = YES	6	(11%)	3	(7%)	0.100	0.195
DF = = 3	Reached target dose =YES Reached Potassium 6 = NO	24	(45%)	30	(70%)		
	Reached target dose =NO Reached Potassium 6 = YES	5	(9%)	1	(2%)		
	Reached target dose =NO Reached Potassium 6 = NO	18	(34%)	9	(21%)		
Reached target dose	Reached target dose =YES	30	(57%)	33	(77%)	0.039	0.048
DF = = 1	Reached target dose =NO	23	(43%)	10	(23%)		
Hyperkalaemia	Reached Potassium 5.6 = YES	26	(49%)	12	(28%)	0.035	0.036
DF = = 1	Reached Potassium 5.6 = NO	27	(51%)	31	(72%)		
Severe Hyperkalaemia	Reached Potassium 6 = YES	11	(21%)	4	(9%)	0.162	0.136
DF = = 1	Reached Potassium 6 = NO	42	(79%)	39	(91%)		

The same analyses were repeated using a 6.0 mmol/L potassium threshold, with its corresponding four-category version. Reaching the potassium threshold (5.6 or 6.0 mmol/L) means the patient recorded that value at least once during follow-up. ITT analysis is a randomisation/permutation test (5000 simulations).

Secondary outcomes—target RAASi dose achievement, hyperkalaemia, and severe hyperkalaemia (all Yes/No)—were analyzed using chi-squared tests (or Fisher’s exact test where appropriate) for PP, and corresponding permutation tests for ITT. Two-sided *p*-values were inferred and seemed as statistically significant if less than 0.05.

### Secondary outcomes

Across the course of the trial, 33 of 43 patients (77%) in the SZC group and 30 of 53 patients (57%) in the placebo group (Table 2) achieved the target RAASi dose at least once; with evidence for a significant between-group difference as supported by the ITT analysis (*p* = 0.048) and the PP analysis (*p* = 0.039). In an analysis for achieving target dose RAASi without reaching a serum potassium of 6.0mmol/L, the study showed a higher proportion of patients in the SZC group reached the target dose compared to the placebo group, as shown in ITT analysis (P=0.036) and PP analysis (P=0.016) (see Table2).

Hyperkalaemia, i.e., serum potassium ≥5.6 mmol/L, occurred in 26 of 53 patients (49%) in the placebo group and 12 of 43 patients (28%) in the intervention group (Table 2), with significant differences observed in both PP and ITT analyses (*P* = 0.035 and *P* = 0.036, respectively). There was no evidence (Table 2) of a significant difference in the proportion of severe hyperkalaemia ( ≥ 6.0 mmol/L) between groups (*P* = 0.162 and *P* = 0.136 in PP and ITT analyses, respectively).

**Fig. 3:**
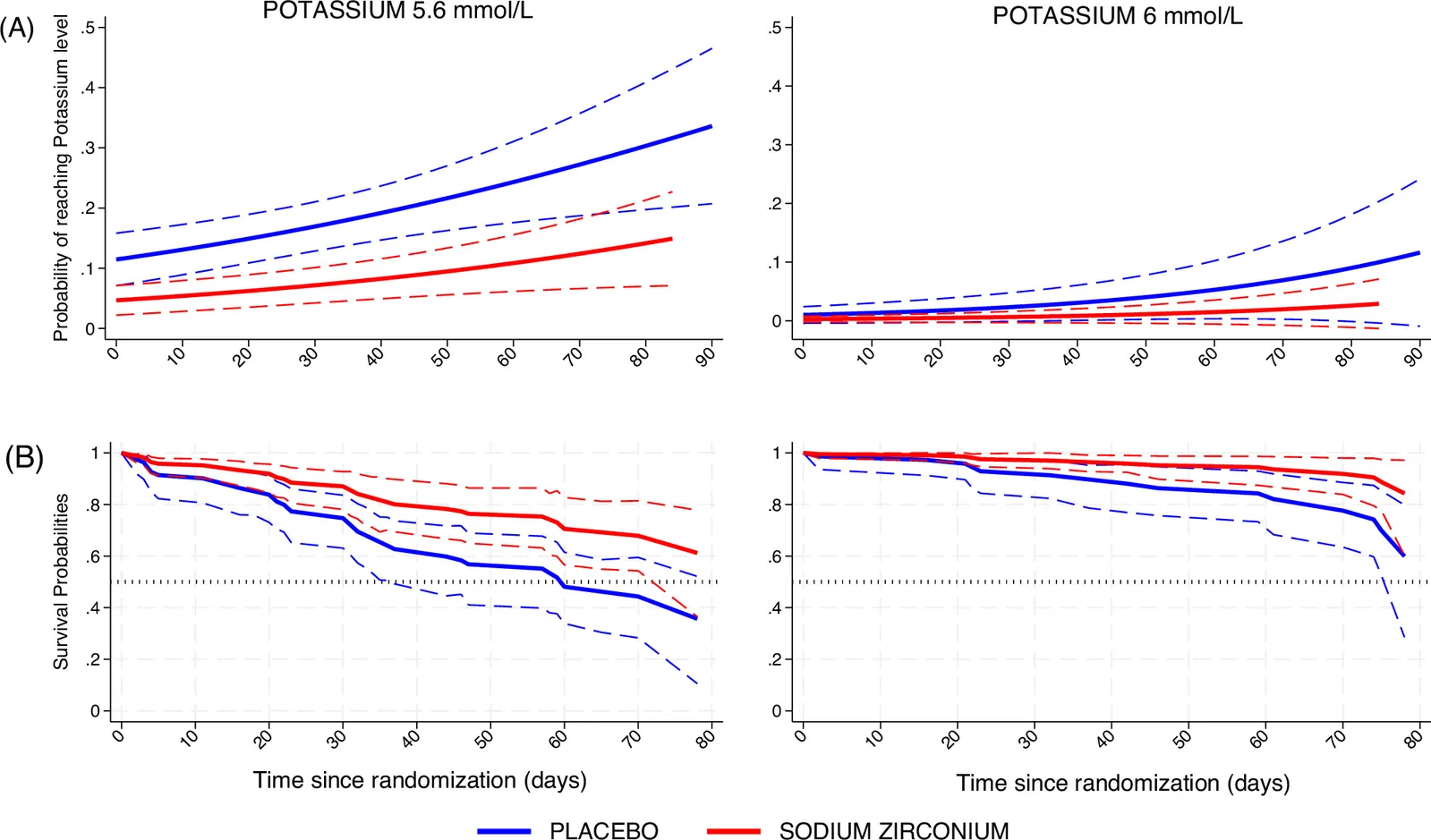
Probabilities of developing hyperkalaemia (>5.5 mmol/L) and severe hyperkalaemia (>6.0 mmol/L). **A** Population-average (GEE-fitted) estimated probability of hyperkalaemia (defined at thresholds of ≥5.6 mmol/L and ≥6.0 mmol/L) across the two intervention groups (*n* = 112), with 95% confidence intervals (dotted lines), during the trial follow-up. Estimates are based on all observed events rather than only the first occurrence during the trial. **B** Survival probability curves for time to first occurrence of hyperkalaemia ≥5.6 mmol/L and ≥6.0 mmol/L, by intervention group (*n* = 112). Curves were derived from Cox proportional hazards models, with 95% confidence intervals (dotted lines) obtained via bootstrapping. Censoring was defined as the date of the last attended study visit or the seventh visit, whichever occurred first, and was assumed to be non-informative. There was no evidence to suggest any violation of the proportional hazard assumption. Two-sided p-values less than 0.05 were considered statistically significant; the values and the sample sizes are shown in Table 5.

GEE longitudinal analyses for hyperkalaemia inferred the probability of its occurrence, accounting for multiple events associated with one participant during the trial and for as long as the patients were followed up, irrespective of finishing the trial (Supplementary Table S3). The probabilities of experiencing these events increased with time (Supplementary Table 3 and Fig. 3A). The odds ratio (OR) of experiencing these events for the Sodium Zirconium group vs. Placebo suggest a strong effect of the intervention on both hyperkalaemia occurrence (OR = 0.38 (95% CI 0.23–0.64)) and severe hyperkalaemia (OR = 0.29 (95% CI 0.10–0.89)) compared to placebo group. Subject-specific inference produced estimates only of slightly different magnitude but qualitatively consistent with the group average (Supplementary Table S3).

Table 3 presents a series of various survival analyses for events defined by hyperkalaemia and its severity, including multiple versions of the log-rank test. The survival analyses for hyperkalaemia included 109 patients; 3 patients (2 in the intervention group, 1 in placebo) withdrew at randomisation and were excluded. Results suggest that patients in the SZC group were approximately half less likely to develop hyperkalaemia ≥5.6 mmol/L (HR = 0.48 (95% CI 0.25–0.91)), with 61% (95% CI 39–75%) surviving event-free beyond day 78, compared to 36% (95% CI 11–53%) in the placebo group (Fig. 3B). For hyperkalaemia ≥6.0 mmol/L, there was marginal evidence (*P* = 0.056) of a reduced risk in the SZC group (HR = 0.33 (95% CI 0.11–1.04)), with 84% (95% CI 63–97%) and 60% (95% CI 28–80%) of patients remaining event-free beyond day 78 in the SZC group and placebo groups, respectively.

**Table 3:** Various survival analyses were performed, including multiple versions of the log-rank test for hyperkalaemia ( ≥ 5.6 mmol/L) and severe hyperkalaemia ( ≥ 6.0 mmol/L)

SODIUM ZIRCONIUM vs. PLACEBO	HR	*p*-value	95%CI LOW	95%CI HIGH	TEST/MODEL
OUTCOME					
Time to Hyperkalaemia 5.6 (days)		0.022			Log Rank
First occurrence		0.020			Log Rank (Wilcoxon–Breslow–Gehan)
		0.021			Log Rank (Peto)
		0.019			Log Rank (Tarone–Ware)
		0.021			Log Rank (Cox regression based)
		0.023			Permutation Log Rank
	0.48	0.025	0.25	0.91	Univariate Cox PH
	0.44	0.023	0.21	0.89	Interval-censored analysis
Time to Hyperkalaemia 6 (days)					
First occurrence		0.047			Log Rank
		0.017			Log Rank (Wilcoxon–Breslow–Gehan)
		0.037			Log Rank (Peto)
		0.024			Log Rank (Tarone–Ware)
		0.042			Log Rank (Cox regression-based)
		0.043			Permutation Log Rank
	0.33	0.059	0.11	1.04	Univariate Cox PH
	0.34	0.070	0.11	1.10	Interval-censored analysis
SENSITIVITY ANALYSES – worst case scenarios for Hyperkalaemia 5.6					
2 SZC patients =early outcome (14 days), 1 Placebo=never		0.066			Permutation Log Rank
2 SZC patients =mid time outcome (45 days), 1 Placebo=never		0.052			Permutation Log Rank
2 SZC patients =late outcome (85 weeks), 1 Placebo=never		0.042			Permutation Log Rank

The classical log-rank test assigns equal weight to events across time; the Wilcoxon–Breslow–Gehan version weights earlier events more heavily; the Peto version uses an alternative variance estimation method; and the Tarone–Ware version weights time points proportionally to the square root of the number at risk, intermediate between the log-rank and Gehan–Breslow approaches.

A sensitivity analysis assigned patients who withdrew after the first visit a small positive survival time (e.g., 0.3). Estimates did not differ meaningfully—unsurprisingly, since none of these patients experienced the event of interest, and survival analyses are primarily driven by event occurrence.

Some discrepancies in inference were observed between the log-rank and Cox proportional hazards results for the outcome defined by severe hyperkalaemia ( ≥ 6.0 mmol/L). Notably, only four patients in the SZC group experienced this event, suggesting that log-rank–based tests, including the permutation version, may provide more reliable inference (*P* = 0.043 is not interpreted as strong evidence) in this case, given the asymptotic nature of inference associated with a Cox proportional model. A sensitivity analysis to these estimates was conducted assuming worst-case scenarios for the patients who withdrew after the first visit (Table 3) – namely two in the SZC group and one in the placebo group. We found that, in a counterfactual scenario in which hyperkalaemia ( ≥ 5.6 mmol/L) occurred early during follow-up in the two patients in the SCZ group with missing data, the difference between the intervention groups was no longer statistically significant (Table 3). Longitudinal analyses of the laboratory measurements taken at each visit are presented in Supplementary Table S3 (for potassium as a longitudinal continuous outcome) and Supplementary Table S4 for eGFR, Sodium, Troponin, NT-pro BNP and ACR.

**Fig. 4:**
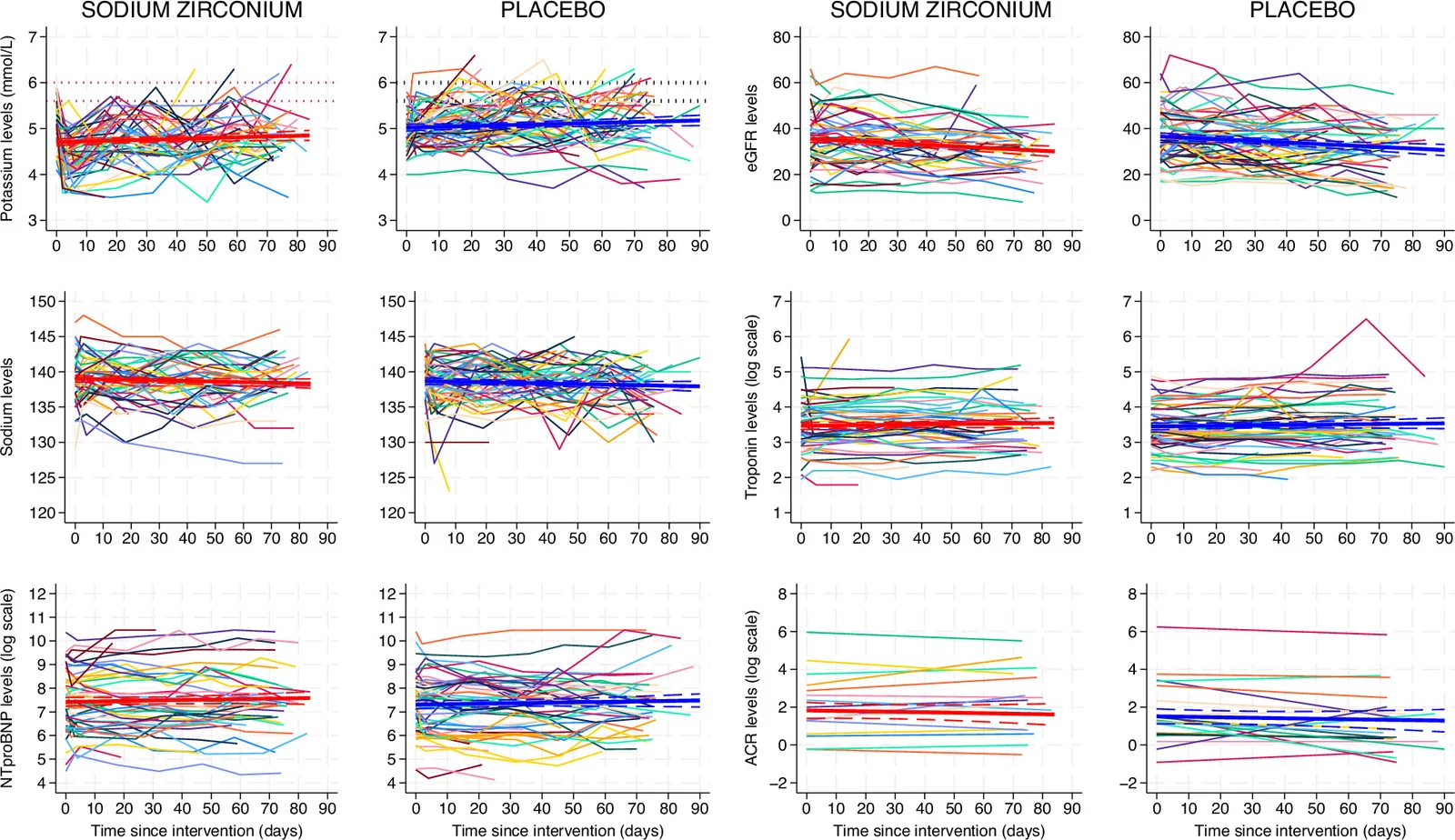
Observed trajectories and model-based mean trends (fixed effects) with 95% confidence intervals over follow-up time, by intervention group (*n* = 112), for potassium (continuous), eGFR, sodium, troponin, BNP, and ACR. Estimates were obtained from mixed-effects (random-effects) models fitted to each longitudinal continuous outcome; troponin, BNP, and ACR were log-transformed, as indicated in the figure. Corresponding estimates, the models’ fitting and the two-sided p-values are presented in Table 4 (potassium) and 6. Potassium levels increased slowly but significantly (*p* = 0.035) over time in both groups; however, mean levels appeared lower in the sodium zirconium group compared with placebo. The analyses are exploratory; hence no multiple comparison adjustment were made.

Potassium levels increased over time, similarly in both groups, by 0.02 mmol/L (95% CI 0.002–0.05) on average every two weeks, with strong evidence (P < 0.001). The average potassium levels across the follow-up were 0.32 mmol/L (95% CI 0.23–0.40) lower in the intervention group compared with the placebo group, adjusted for follow-up time (Supplementary Table S3 and Fig. 4). The fortnightly rate of change in potassium levels was 0.03 mmol/L; 95% CI 0.001–0.06, but there was no evidence to suggest a difference between the two groups (*P* = 0.134) after adjusting for baseline values.

**Fig. 5:**
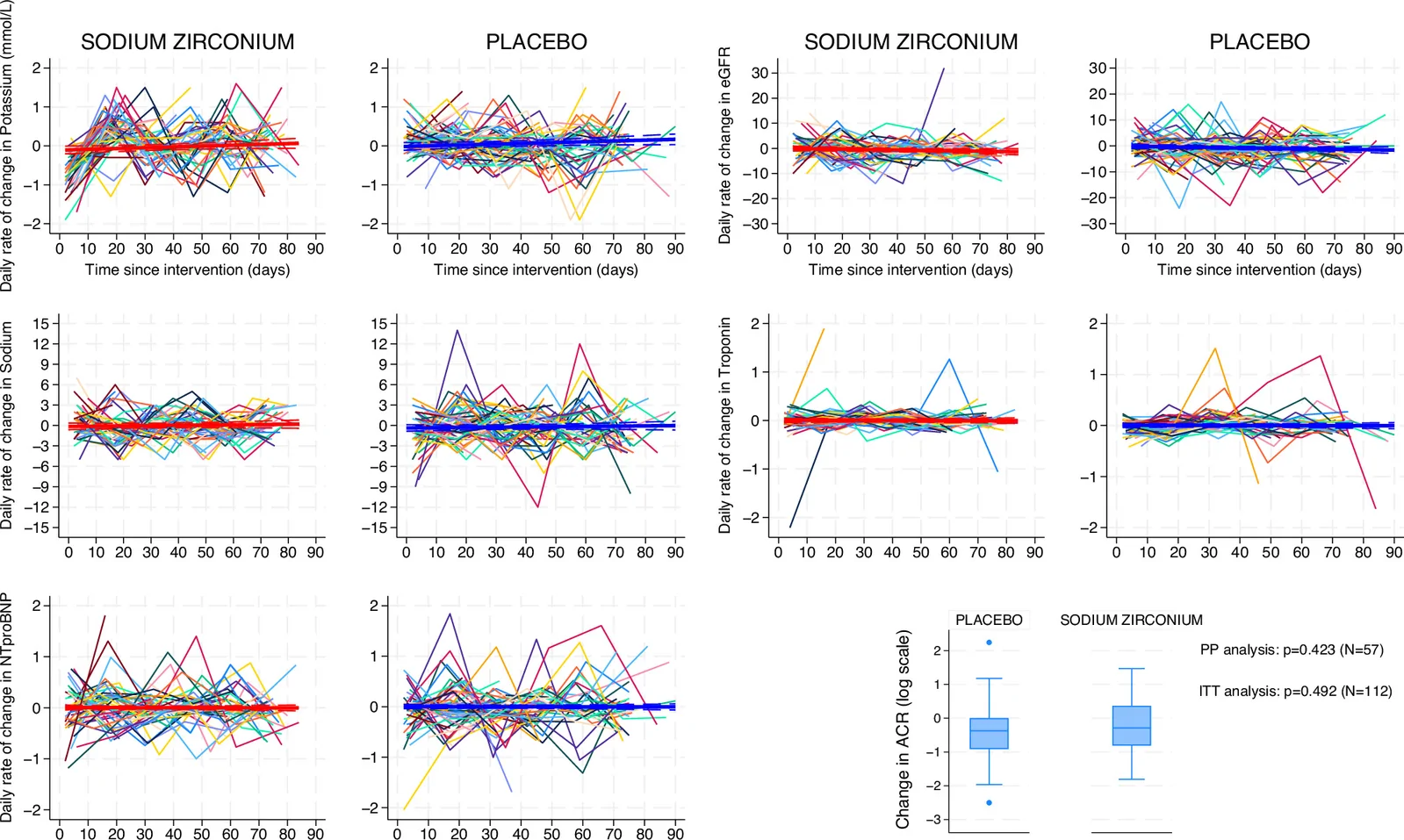
Individual trajectories and model-based mean trends (fixed effects) with 95% confidence intervals over time, by intervention group, for potassium (continuous), eGFR, sodium, troponin, BNP, and ACR. Estimates were obtained from mixed-effects (random-effects) models fitted to each longitudinal continuous outcome, as indicated in the figure. The estimated time slopes are reported in Supplementary Table S6. All the analyses are conducted under missing at random assumption on missing observations. For ACR, only two measurements per participant were available (at randomisation and at the end of follow-up), and only 57 participants (51%) had both measurements. The boxplots of the observed ACR measurements at the two time points depict the medians, the first and the third quartiles (lower and upper hinge of the box), lower and upper adjacent values (whiskers ‘ends) and outside values. The intention-to-treat analysis, based on a permutation test and all 112 patients, was consistent with the per-protocol analysis. Two-sided p-values less than 0.05 were considered statistically significant.

Supplementary Table S4 does not reveal evidence of differences between groups in the levels eGFR, Sodium, Troponin, NT-pro BNP and ACR levels during the trial follow-up. The eGFR declined over the follow-up period by 0.92 mL/min/1.73 m² (95% CI 0.43–1.41) every two weeks. The rate of change in eGFR also decreased with higher baseline eGFR values, by 0.05 mL/min/1.73 m² (95% CI 0.01–0.09) for each one-unit increase in baseline eGFR; that is, participants with higher baseline eGFR values experienced a smaller rate of decline during follow-up but no evidence of a difference between the two groups is inferred. Supplementary Table S4 also presents the estimated baseline means (or geometric means for log-transformed variables) across intervention groups. Average changes across the two groups are presented in Fig. 5.

The specific RAASi agents used are summarised in supplementary table S5. There was no evidence to suggest support for differences in dose escalations between the two groups (Supplementary Table S6).

Supplementary Tables S7 and S8 investigated patterns in patients’ withdrawal and missing observations. In the placebo group, 3(5%) of the participants withdrew based on some clinical decision and the same number based on their own decision. In the intervention group, 2(4%) of the participants withdrew based on some clinical decision and 8(15%) based on their own decision. There was no evidence of a difference in the distribution of attrition numbers between the two groups (*P* = 0.209).

Binary longitudinal data analysis (Supplementary Table S8), with a response indicating patient’s missing visit or otherwise, did not reveal patterns in the missing data by baseline characteristics except for sodium levels. It seems that attrition was less likely for participants with higher levels of sodium at baseline (*p* = 0.002). The number of admissions in the relatively short follow-up was only 14 in 13 participants. Two in 43 patients (4.7%) participants on sodium zirconium and 11/54 (20%) participants on placebo were admitted to hospital, and the *p* = 0.034 values indicated some evidence of a significant difference between the groups. There was one admission due to heart failure in the intervention group and three in the placebo group. The reasons for admissions are shown in supplementary Table S9.

**Fig. 6:**
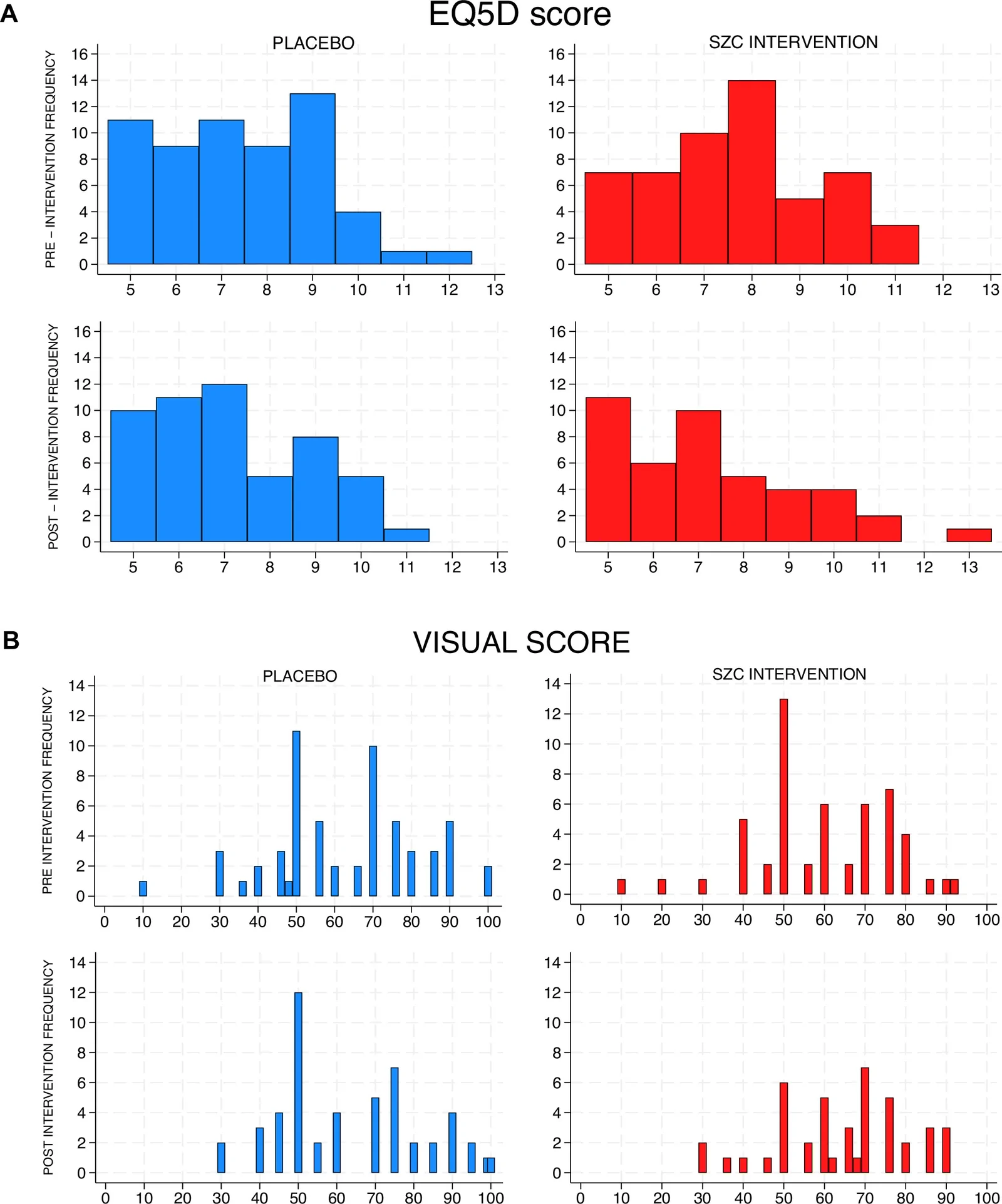
Quality of life scores in placebo and sodium zirconium groups before and after the trial period. **A** EQ5D scores distributions by intervention group (*n* = 112), before and after the trial raw data shown. Corresponding estimates, the sample sizes and the two-sided p-values are presented in Table 7. **B** Visual scores distributions by intervention group (*n* = 112), before and after the trial raw data shown. Corresponding estimates, the sample sizes and the two-sided p-values are presented in Table 7.

There was no evidence to support a difference in quality of life between or within the randomised groups with ITT analysis (Table 4, Fig. 6A, B).

**Table 4:** Quality of life data analysis: summary of EQ-5D and visual analogue scores stratified by intervention group

		Placebo		Intervention		Placebo	Intervention	POST		CHANGE	
		PRE	POST	PRE	POST	INTRA GROUP CHANGE		PP	ITT	PP	ITT
						POST-PRE	POST-PRE	INTER GROUP		INTER GROUP	
EQ5D score	N	59	52	53	43	52	43	95	112	95	112
	Mean/SD	7.4/1.8	7.2/1.7	7.7/1.7	7.3/2.02	-0.25/1.6	-0.23/1.4				
	Median(Q1-Q4)	7(6-9)	7(6-9)	8(6-9)	7(5-9)	0(-1, 0.5)	0(-1, 1)				
	Min-Max	5-12	5-11	5-11	5-13	(-4, 5)	(-3, 3)				
	*P*-values					0.23	0.21	0.97	0.52	0.07	.45
Visual score	N	59	51	53	43	51	43	94		94	
	Mean/SD	62.6/19.1	64.0/18.8	59.1/17.1	64.3/15.6	1/16.9	3.8/14.5				
	Median(Q1-Q4)	65(50-75)	60(50-75)	60(50-75)	65(50-75)	0(-10, 10)	5(-5,10)				
	Min-Max	10-100	30-100	10-92	30-90	(-50, 45)	(−20, 45)				
	*P*-values					0.63	0.16	0.78	0.85	0.19	0.92

Inference covers intra- and inter-group differences for end-of-trial outcomes (POST analyses) and end-versus-baseline differences (CHANGE analyses). Both PP and ITT analyses were performed for post EQ-5D and visual scores, and for within-group changes (last visit minus first visit).

## Discussion

This phase 3, double-blind, randomised controlled trial demonstrated that treatment with the potassium binder SZC, in conjunction with close monitoring, enabled HFrEF patients to achieve RAASi, including mineralocorticoid receptor antagonists, ACE inhibitors, angiotensin receptor blockers, or angiotensin receptor–neprilysin inhibitors. During RAASi up-titration, SZC was associated with a lower incidence of hyperkalaemia and a longer time free from hyperkalaemia, without an excess of adverse events. Severe hyperkalaemia was also less likely in the SZC group.

Our protocol was similar to the approach used in the Safety, Tolerability and Efficacy of up-titration of guideline directed medical therapies for acute heart failure (Strong-HF): a multinational open label RCT study, which evaluated rapid up-titration of guideline-directed medical therapy, including RAAS inhibitors, in patients with acute heart failure; treatment was escalated from half to full dose over two weeks, with safety assessments at weeks 1, 2, 3, and 6^11^.

Notably, baseline use of ACEi/ARB/ARNI and MRA was high (64% and 94%, respectively), and overall kidney function was better than in our cohort (baseline eGFR in intervention arms 61.92 (19.92) versus 34.72 (11.96) ml/min/1.73m2 in our study).

In our study, RAAS inhibitor doses were increased in a stepwise manner from the starting dose to the half-maximal dose and subsequently to the maximum dose at two-week intervals, provided patients did not develop hypotension, hyperkalaemia, or treatment intolerance. Consistent with the Strong-HF protocol, safety monitoring included clinical review and measurement of electrolytes, BNP and creatinine at each visit.

Importantly, despite the greater clinical complexity and more advanced renal impairment in our population, this up-titration strategy was both feasible and well tolerated. There was no evidence to suggest a shift in the quality of life between treatment arms in our study (See Table 7). In Strong-HF, quality of life improved at 90 days in the high-intensity treatment group.

Adverse events, including oedema, hypotension and gastrointestinal symptoms, were common but did not differ between the SZC and placebo groups. A substantial proportion of patients in both groups discontinued the study; however, withdrawal rates were comparable between the two groups, and similar to those reported in a recently completed trial of SZC for maintaining more than half the target dose of a mineralocorticoid receptor-antagonist in HFrEF patients, i.e., peripheral oedema^9^. Based on our data, there was no evidence to support treatment-related changes in serum sodium concentrations or in cardiac biomarkers, including NT-proBNP and troponin. One patient in the SZC group developed hypokalaemia (3.4 mmol/L) at the time of the 5^th^ visit, but subsequently recovered with values of 4.8 mmol/L (6^th^ visit) and 4.7 mmol/L (7^th^ visit – the last).

The fear of hyperkalaemia in patients with chronic kidney disease often hinders the initiation and up-titration of RAASi, despite their proven benefits in managing concomitant heart failure. For example, hyperkalaemia ( > 5 mmol/L within 7 days of hospital admission) was linked to reduced RAASi use and higher 180-day mortality, which may be preventable with potassium binders; as demonstrated in this study^12^. In patients with chronic kidney disease (CKD) stages 3 to 5—such as those in our cohort (mean eGFR 34 mL/min/1.73 m²)—the risk of discontinuing renin-angiotensin-aldosterone system inhibitors (RAASi) was elevated, primarily due to hyperkalaemia (within 90 days of potassium>5.0 mmol/L)^13^. This may ignite the necessity of using of potassium binders to maximise and maintain RAASi therapy and mitigate associated cardiorenal risks as achieved in our study.

A recent randomised trial investigated patients with heart failure and reduced ejection fraction, a significant proportion of patients also had chronic kidney disease (CKD), defined as eGFR <60 ml/min/1.73 m² (53% in the placebo group and 73% in the SZC group). The study examined whether the use of SZC allowed for the continued administration of target dose, defined as more than half-dose, mineralocorticoid receptor blocker for six months, compared to placebo and maintain a serum potassium 3.5-5.0 mmol/L^9^. In the study with SZC time to hyperkalaemia was reduced (HR 0.51 95% CI 0.37– 0.91) very similar to the present study (HR 0.48 95% CI 0.25– 0.91). The optimal response of achieving target dose of mineralocorticoid receptor was 71% in the SZC and 36% in placebo. In the present study target doses of both mineralocorticoid receptor antagonist and either ACE inhibitor, angiotensin receptor blocker or angiotensin receptor and neprilysin inhibitor was 56% in SZC and 28% in placebo. However, all patients in the present study suffered from chronic kidney disease, including patients with eGFR <30 ml/min/1.73 m^2^, and the definition of hyperkalaemia was a serum potassium level of more than 5.6. mmol/L as opposed to 5.0 mmol/L in the compared study.

In an initial trial in 105 heart failure patients with CKD or history of hyperkalaemia, the maximal dose of spironolactone, i.e., 50 mg daily, could be maintained in 91% patients with serum potassium less than 5.5 mmol/L with RLY5016 versus 74% (*p* = 0.019) with placebo, but the eGFR at baseline in the intervention arm was higher than in the present study 84 ± 35 ml/min/1.73m^2^ ^14^.

A larger trial of heart failure and CKD the same agent, patiromer, while maintaining half maximal dose of ACEi/ARB/ARNI maximal dose of spironolactone or eplerenone i.e 50 mg daily, was maintained in 86% versus 81% with placebo (HR 0.62; 95% CI 0.45, 0.87; *P* = 0.006) in maintaining normokalaemia however the eGFR at baseline was 62.6 ± 22.6 ml/min/1.73m^2^ ^15^.

The strength of the study comes from its, unique trial population of elderly patients with advanced kidney disease. Mean kidney function was 35 ± 12 ml/min/1.73m2, and age was 73 (38-92) years in our LIFT study as opposed to 58 ± 19 ml/min/1.73m2 and 70 (36-95) years in above study, Realise-K. Elderly and often frail people were able to participate in this rigorous trial without evidence of worsening of quality of life (as measured by EQ5D and visual scores) during the trial yet benefitted from the rapid up-titration of proven therapy with close monitoring of clinical and biochemical parameters.

The trial has some limitations. This was a single centre study with clinicians following these patients with clinical and biochemical examinations every two weeks allowing the rapid up-titration of RAASi which may not be feasible in standard clinical practice. The follow-up was short, and hence, the long-term effects of the potassium binder therapy for RAASi maximization require further investigation. The primary outcome of the study was RAASi maximization; however, the long-term clinical benefits in these chronically ill advanced CKD patients with HFrEF will require a larger and longer trial. The cost of potassium binders in economically challenged healthcare settings may prohibit the implementation of the benefits of the potassium binders as shown in the study.

The sex distribution observed in our sample is consistent with that reported in a recent CKD–HF clinic cohort and remains plausible when further restricted to patients with reduced ejection fraction, supporting the representativeness of our study population^16^.

In older patients with heart failure with reduced ejection fraction and moderate to advanced chronic kidney disease, sodium zirconium cyclosilicate enables the rapid up-titration of mineralocorticoids and ACE-inhibitor or angiotensin-receptor-blocker or angiotensin-receptor-neprilysin-inhibitor, without excess adverse effects.

## Methods

### Study design

We conducted a phase III, double-blind, parallel arm randomised controlled trial in patients with chronic kidney disease and heart failure, with participants allocated in a 1:1 ratio. The study protocol, which outlines the study design, primary and secondary outcomes, and the input and rationale for the sample size calculation, has been published elsewhere^10^. A succinct summary of trial elements is presented below. The study was registered with clinicaltrials.gov (NCT05004363) and EudraCT (2020-002946-18). The Consort checklist can be found in Supplementary Note 1.

### Patients

We enroled adult patients ( ≥ 18 years) with heart failure and reduced ejection fraction (NYHA class II–IV) and chronic kidney disease, defined as a stable estimated glomerular filtration rate (eGFR) < 60 mL/min/1.73 m² after providing a written informed consent between 1 February 2022 and 1 July 2022. Eligible patients were required to have a serum potassium concentration 5.0-5.5 mmol/L within three months prior to screening, a systolic blood pressure >90 mmHg without clinically significant postural hypotension, and to be receiving either no or submaximal doses of RAASi. Patients were excluded if they were pregnant, had an active malignancy or infection, a recent acute coronary syndrome, were receiving potassium supplementation, or demonstrated a prolonged QT interval on electrocardiography. Initially, individuals with a body mass index (BMI) > 35 kg/m² were excluded; this threshold was subsequently revised to >40 kg/m². Similarly, patients with glycated hemoglobin (HbA1c) > 70 mmol/mol were originally excluded, but this criterion was later refined to exclude those with persistently uncontrolled glycaemic status and poor treatment adherence. All patients were recruited from the cardiology, nephrology, and cardiorenal clinics and were ambulatory at baseline. All included patients underwent an echocardiogram or an MRI scan to diagnose HFrEF with EF ≤ 40%. Our population study was unique in that it included patients with advanced chronic kidney disease with eGFR<30 and, therefore, were very prone to AKI and hyperkalemia.

### Intervention

The RAASi optimisation treatment period ranged from 2 to 12 weeks, depending on the patient’s baseline RAASi dose, with follow-up extending from 4 to 14 weeks. Data were collected at each scheduled study visit. The investigational medicinal product (IMP) was self-administered at home in 5- or 10-gram sachets, in accordance with a standardised protocol^2,10^. At each visit, potassium levels were assessed using both an urgent point-of-care test and a standard laboratory measurement. Patients with serum potassium of 3.5 – 5.0 were treated with 5 g daily of IMP, while treatment was withheld when serum potassium was <3.5 mmol/L and increased to 10 g daily when potassium exceeded 5.0 mmol/L, as detailed in Table S1. Patients with a potassium level >6.0 mmol/L on point-of-care testing, confirmed by urgent laboratory measurement, were immediately referred from the clinical research facility to the on-site emergency department for management of hyperkalaemia.

At each study visit, two weeks apart, the treating physician—blinded to treatment allocation—was instructed to up-titrate RAAS inhibitors according to the study protocol, progressing from the starting dose to the half-maximal dose and subsequently to the maximum dose at two-week intervals, provided it was safe to do so on clinical and biochemical evaluation (Table S2). Up-titration was undertaken provided the patients were not hypotensive (systolic blood pressure <90 mmHg), had no symptomatic or clinically significant postural drop in systolic blood pressure ( ≤ 20 mmHg), were not hyperkaliaemic, and had not experienced significant adverse effects at the previous dose. Table S3 outlines the recommended dose escalation for patients not receiving any RAASi inhibitors at baseline and Table S5 shows the specific RAASi agents used.

The primary objective of this study was to compare the proportion of patients in the SZC group versus the placebo group who achieved the target RAASi dose while maintaining a serum potassium concentration below 5.6 mmol/L.

### Outcomes’ Definitions

The primary endpoint was defined as a composite binary outcome, with success defined as achieving both the target RAASi dose and maintaining a serum potassium concentration less than 5.6 mmol/L throughout the trial. Failure was defined as either not achieving the target RAASi dose or achieving it in the presence of a serum potassium concentration reaching 5.6 mmol/L or higher.

A refined four-level categorical response was defined by jointly classifying the target dose achievement (YES or NO) and hyperkalaemia status (serum potassium ≥5.6 mmol/L or <5.6 mmol/L).

Three other secondary univariate binary outcomes were defined for (i) achievement of the target RAASi dose or not, (ii) first occurrence of hyperkalaemia (serum potassium ≥5.6 mmol/L) or not, and (iii) first occurrence of severe hyperkalaemia (serum potassium ≥6.0 mmol/L) or not.

Their longitudinal versions, of these outcomes were also considered for hyperkalaemia (serum potassium ≥5.6 mmol/L) or not, and hyperkalaemia (serum potassium ≥6 mmol/L) or not, to investigate all events of the same type occurring during the follow up.

Longitudinal biochemical outcomes (continuous variables) comprised repeated measures of serum potassium, eGFR, sodium, troponin, NT-proBNP, and urine albumin-to-creatinine ratio (ACR) during the trial follow up. Their change were calculated as differences from the preceding measurement, enabling rate-based interpretation. Log-transformed values were used when appropriate to approximate normal distribution. Quality of life scores and their changes were also evaluated using European Quality 5-Dimensions (EQ5D) and visual scores, at randomisation and at the last visit^17^.

### Randomisation

Eligible patients were enroled by study clinicians and subsequently assigned to treatment groups by the study pharmacist according to a 1:1 allocation sequence generated using computer-based random numbers (simple randomisation). The allocation sequence was concealed from the enroling clinicians and patients, and treatment assignment was implemented by the pharmacist to ensure allocation concealment. The investigational product SZC and placebo were identical in packaging, appearance, taste, smell, and weight. Randomisation list was produced in Stata and allocation was implemented by the research pharmacist. Because the trial closed before the originally planned sample size of 130 participants was reached, a small numerical imbalance between groups is expected. Importantly, baseline characteristics remained broadly comparable between the two groups, consistent with the integrity of the randomisation process.

### Sample Size calculation

Group sample sizes of 52 will achieve approximately 90% power to detect a difference between the group proportions of 0.3 using a two-sided Z-test with pooled variance and a two-sided χ^2^ test. The proportion of responders in the placebo arm is assumed to be 0.5, with the corresponding proportion in the active arm 0.8 under the alternative hypothesis. The significance level (α) of the test is 0.05. This results in a total of 104 patients required and, accounting for 20% loss to follow-up, we will recruit 130 patients to be randomised to each intervention group. Further details can be found in the published protocol^10^.

### Statistical analytical methods

Data were unblinded only after trial completion, cleaned, curated, and prepared for analysis with reference to assessment notes where necessary. Baseline characteristics, including laboratory values, are summarised by treatment group. Categorical variables are reported as counts and percentages, and continuous variables as means (SD) or medians (IQR). For binary and categorical univariate outcomes (the primary binary outcome and its refined version, the one indicating reaching target dose or not, hyperkalaemia and severe hyperkalaemia), treatment effects were first evaluated using per-protocol (PP) analysis using chi-squared **χ²** or Fisher’s exact tests.

Permutation versions of these tests were applied for the ITT analyses. The advantages of using permutation tests for ITT include: no need for large sample approximations, respects randomisation and works well when sample sizes are small and groups are imbalanced.

We have also estimated the odds ratios for the effect of RAASi on the primary outcome, using multiple imputation techniques for the observed data analysis^18^.

To account for the multiple observations for a participant, associated with measurements on the biochemical markers during the duration of the study (potassium, eGFR, sodium, troponin, NT-proBNP, and ACR), mixed-effects longitudinal models, including random intercepts and random slopes for time with an unstructured variance–covariance matrix, were fitted. These models were applied to both absolute values and changes over time for these markers, as defined in the Outcomes section. Parameters were estimated using maximum likelihood under MAR assumption for missing observations.

The randomisation process produced two intervention arms with no statistically significant differences in terms of baseline characteristics. Hence, each longitudinal outcome model associated with a biochemical marker included the intervention group and its interaction with time as potential covariates. This has been done with two purposes: to investigate inter-group differences and groups’ patterns with time which can be different across groups. If the interaction term was not statistically significant, it was excluded from the final models. However, analyses of longitudinal changes (namely the values representing the difference between the current and previous values) were additionally adjusted for corresponding baseline (first visit) marker values to improve estimation efficiency^19^. ACR was measured twice only, at randomisation and at the patient’s last visit.

Longitudinal binary outcomes were analysed using both subject-specific effects using mixed-effects logistic regression models with random intercepts and slopes for time and unstructured setting for the covariance (variance–covariance) using maximum likelihood estimation and population-averaged effects using generalised estimating equations (GEEs). Subject-specific estimates reflect within-individual changes in outcome odds, whereas population-averaged estimates represent mean changes at group level across the trial^20^. These approaches may differ quantitatively, and they differ in their assumptions about participants' attrition—missing at random (MAR) for mixed models and missing completely at random (MCAR) for GEEs—which are not empirically verifiable^18^.

Using a similar argument as for continuous longitudinal outcomes, given the intervention groups statistical equivalence, models’ covariates included the intervention arms indicator and their interaction with time.

Given the intervention groups statistical equivalence, models’ covariates included the intervention groups and their interaction with time – that has not been observed to be statistically significant hence we did not keep the terms in the model. Associations between the outcomes and explanatory variables were assessed by odds ratios (ORs) and their 95% CIs.

Time-to-event outcomes were analysed for both hyperkalaemia and severe hyperkalaemia. Log-rank tests, including Wilcoxon and Peto variants (weighting the analyses towards early or late events, respectively) and their permutation-based counterparts, were used to compare time-to-event distributions across the intervention groups. Cox proportional hazards models were also fitted on intervention group variable only, with proportionality assumptions formally assessed^21^. Associations between outcomes and group variable were measured by hazard ratios and their corresponding 95%CIs.

Interval-censored survival models were also used, as hyperkalaemia events could only be realistically captured within the follow-up intervals. Recurrent hyperkalaemia events were analysed using survival models accounting for within-participant variability. The number of RAAS inhibition medication escalations per participant was modelled as a Poisson count, with the total time spent in the trial included as an offset to investigate whether the rates of escalation differed by intervention groups. Negative binomial regression was used to assess potential overdispersion which has not been observed.

Patterns of missing observations at the follow-up were examined across treatment groups using a multilevel logistic regression for a longitudinal binary outcome, where 1 indicates a missing observation and 0 indicates an observed value. Survival probability curves by intervention groups and their uncertainties were derived for both first occurrence of the hyperkalaemia ( ≥ 5.6 mmol/L) and severe hyperkalaemia ( ≥ 6 mmol/L), respectively. The curves are predictions following a Cox proportional hazard regression fitted to the data with bootstrapping used for the 95% CIs. Censoring is defined as the last date the participant attended the visits or the date of the 7^th^ visit, whichever came first. The censoring time distribution is assumed to be independent of that of time to event distribution (non-informative censoring) and there was no evidence for a violation of the proportional hazards’ assumption.

These models were applied to both absolute values and changes over time, as defined in the Outcomes section. Parameters were estimated using maximum likelihood under the MAR assumption for missing observations. All models included the intervention group and its interaction with time; however, as the interaction term was not statistically significant, it was excluded from the final models. Analyses of longitudinal changes were additionally adjusted for baseline (first visit) values of the corresponding outcome to improve estimation efficiency.

Stata 18 (StataCorp, 2023. Stata Statistical Software: Release 18. College Station, TX: StataCorp LLC) has been used for data analysis and graphs.

### Trial monitoring

The study received ethical approval from the Cambridge NHS Research Ethics Committee 20/EE/0227 and the Medicines and Healthcare Regulatory Authority (CTA 12853/0009/001-0001).

Trial oversight was ensured through a predefined governance structure. The study was conducted as a double-blind trial, with treatment allocation concealed from all investigators, including the study statistician, until completion of data collection and database lock. Interim analyses were pre-specified in the protocol and undertaken after approximately 10%, 50%, and 75% of participants had completed follow-up^10^. These analyses were performed on blinded data and focused on feasibility, tolerability, safety signals, and data completeness, without unblinding treatment allocation.

An independent Data Monitoring Committee (DMC), comprising two nephrologists and one cardiologist, provided oversight throughout the trial. A Trial Management Committee, chaired by an independent clinician and including senior investigators, met periodically to review trial conduct. In addition, the study management team met quarterly to assess recruitment, protocol adherence, safety, and data quality. Data were collected using secure electronic case report forms and were accessible only to authorised personnel. The study was conducted in accordance with Good Clinical Practice and was subject to monitoring, audit, and inspection by authorised representatives of the sponsor, host institution, and regulatory authorities.

The Data Monitoring Committee met every three months during the trial to review recruitment, protocol adherence, safety considerations, and data quality, in line with the protocol. The sponsor reported all adverse events to the UK Government Medicines and Healthcare Products Regulatory Agency and the funder. The Trial Steering Committee (TSC) met every 6 months to monitor trial progress, patient safety, research governance, the protocol, and DMC reports. The trial management committee met every 7 to 30 days to discuss recruitment and other issues regarding the running of the trial and implement the changes suggested by TSC and DMC if necessary.

### Reporting summary

Further information on research design is available in the Nature Portfolio Reporting Summary linked to this article.

## Supplementary information

Supplementary information

Reporting Summary

Transparent Peer Review file

## Data Availability

The study protocol has been published elsewhere^10^. All study data supporting the findings of this study are available in the article and supplementary information file. Individual participant data are not publicly available due to patient privacy and confidentiality obligations. Deidentified individual participant data that underlie the results reported in this article can be made available to other qualified researchers who provide a methodologically sound proposal via a request to the corresponding author (Debasish.banerjee@stgeorges.nhs.uk). The dataset will be provided in compliance with applicable UK data protection law (UK GDPR) and requirements for consent and anonymisation, after full publication of the study and related work. Requests will be reviewed and a response provided within 3 months. A signed data access agreement with the study sponsor is required before data are shared, and use will be restricted to the purpose specified in the approved proposal.
