# Supplementary material for: Rapid Renin Angiotensin Aldosterone System inhibitor optimisation in patients with chronic kidney disease and heart failure with reduced ejection fraction using potassium binder: a randomised, double-blinded, placebo-controlled trial
Source: Nat Commun. 2026 Oct 14;17:11614. doi: 10.1038/s41467-026-78377-0 (PMC13754891; doi:10.1038/s41467-026-78377-0)
Supplement: Supplementary file 2 [file 41467_2026_78377_MOESM3_ESM.pdf]

nature portfolio

Peer Review File

Rapid Renin Angiotensin Aldosterone System inhibitor optimisation in patients with chronic kidney disease and heart failure with reduced ejection fraction using potassium binder; a randomised, double-blinded, placebo-controlled trial

Corresponding Author: Professor Debasish Banerjee

This file contains all reviewer reports in order by version, followed by all author rebuttals in order by version.

Version 0:

Reviewer comments:

Reviewer #1

(Remarks to the Author)
Nature Communications Manuscript Review of Manuscript#: NCOMMS-25-97999-T

Summary
The investigators conducted a double-blind, placebo-controlled, randomised trial to determine whether sodium zirconium cyclosilicate (SZC) enables renin-angiotensin-aldosteone inhibitors (RAASi) up-titration in patients with stage 2-5 chronic kidney disease (CKD) and reduced ejection fraction heart failure (HFrEF) and a serum potassium of 5.0–5.5mmol/L over 14 weeks. 112 patients were randomized to SZC (n=53) or placebo (n=59). Patients in the SZC group were 3 times more likely to reach the target dose without reaching potassium 5.6mmol/L (OR= 3.16; 95% CI 1.35–7.39; P=0.008), than in the placebo group - intention-to-treat analysis (ITT). Hyperkalaemia occurred in 28% of SZC patients compared to 49% with placebo (P=0.036 ITT analysis (HR=0.48; 95%CI 0.25–0.91). The trial did not find significant differences between two groups in quality of life, biomarkers or adverse events. SZC appears to safely support rapid RAASi optimisation in HFrEF patients with CKD.

Critique
Achieving guideline directed medical therapy in patients with HFrEF or advanced CKD or both is important for reducing risk for kidney failure and cardiovascular death. However, as the investigators point out hyperkalemia is a major obstacle to achieving GDMT in these patient populations because of their underlying diseases and the need for RAASi as part of the GDMT. Many real world studies observational studies have used administrative data to determine whether potassium binders enable use of RAASi and the findings suggest that their use is effective. However, few studies have carefully examined the use of potassium binders, including SZC, to improve clinician's ability to optimize GDMT in high risk populations such as those with stages 3-5 CKD and documented HFrEF. The investigators are to be commended for conducting a randomized double-blind placebo controlled trial to address this issue. While the findings support their conclusion the trial was 14 weeks in duration and could not answer the more pressing question of whether enabling use of RAASi makes a difference in clinical outcomes including kidney failure, heart failure and mortality. And, unfortunately, there are numerous concerns with the study design and conduct that raise serious concerns about the conclusion as detailed below.

Strengths
• The patient population under study is a strength. This is a complicated and high risk group of patients that need to be studied carefully as the investigators recognize
• The double-blind placebo controlled design is another strength
• The use of SZC as a test agent is another strength as it has proven efficacy to lower potassium in patients with heart failure, kidney disease, or both and is well tolerated by patients.
• Evaluation of patient reported outcomes is a strength

Weaknesses

Major

• The major weakness of this manuscript is that it provide no guidance for clinicians on how to uptitrate RAASi. While I believe that they have provided sufficiently robust data about the enabling effect of SZC on uptitration of RAAS (with some reservation given the discordant number of participants completing the trial in the SZC vs placebo) group, they do not explain how the uptitration was done let alone the rate at which uptitration was successfully achieved. So, as a clinician I can believe the data but it is no help to me in managing the patient. What the investigators have shown is a proof of concept but it is of little or no clinical value to the broader community of clinicians caring for these very chronically ill and highly vulnerable patients.

• The conclusion should be amended or the investigators should illustrate rapid titration, that is how quickly or slowly the RAASi was titrated. The only thing I can find regarding "rapid" uptitration is that they protocol called for titrating on a 2 week basis and this was done in a 14 week trial. But the investigators cite no literature that defines robustly what "rapid" uptitration means. To put this in the title of the paper and in the conclusion with no scientific evidence to support it, seems unconvincing to me.

• In review of the LIFT protocol, it is not clear that all participants had an echocardiographic or cardiac MRI to confirm HFrEF. The inclusion criteria from LIFT states that Heart failure was diagnosed on clinical grounds or echo confirmed. It seems that they only required an echo for inclusion if the potential participant had atrial fibrillation. How do the investigators know that a patient with :clinical" diagnosis of heart failure has HFrEF vs HFpEF?

• The manuscript should include the definition of the target dose of RAASi and not just refer to the LIFT trial protocol. The LIFT publication does not explicitly state how the target dose of RAASi was achieved. The study flow char in Fig 1 of the paper only describes use of spironolactone and ramipril but no other RAASi that participants are permitted to use. This is important for three reasons: 1) the primary outcome of the study is the proportion of patients in SZC vs placebo who achieved the target RAASi dose while maintaining a serum potassium below 5.6 mmol/L and 2) to understand how the target RAASi and 3) to understand whether multiple algorithms were used, for example tailored to the patient depending on the severity of HFrEF and/or CKD. None of this is specified in the Methods section of this manuscript.

• The Methods section does not explain where the participants were recruited from. Were participants recruited from cardiology, nephrology, general medicine clinics? Were hospitalized patients eligible for the study?

• Further, the investigators allude to a semi-automated Stat script to tailor final RAASi dosing but they do not provide the specifics. This is critically important to analyzing the results of the study and particularly important if the findings are to be translated into clinical practice. Otherwise, how can a clinician use the data from this manuscript to help their patients?

• They state that separate binary outcomes were also specified for RAASI dose achievement and occurrence of hyperkalemia including a separate one for severe cases, but they do not define "severe cases". What does severe mean in this context?

• The manuscript notes that patients provided informed consent but they do not indicate whether or not the protocol was approved by an institutional review board or ethics committee.

• The manuscript does not state whether a data monitoring committee (DMC) was involved in the trial and if so what their role was in monitoring the conduct of the trial. They state that the study was not unblinded until it was completed suggesting that no one including a statistician was involved in viewing the data. While I understand this is not a hard clinical outcome trial many institutions require a DMC for a multisite study especially in such a high risk population. Even many single center studies employ a DMC.

• I find the statistical methods very confusing. The investigators do not state clearly what statistical method was used for the primary outcome. Instead, they make general statements about analyses, for example: "For binary and categorical univariate outcomes, treatment effects were first evaluated in the per-protocol (PP) analysis using χ^2 or Fisher's exact tests. In the intention-to-treat (ITT) analysis we used the permutation-based versions of these tests, which preserve the randomization structure of the data and provide exact inference without the need for distributional assumptions. We have also estimated the odds ratios for the effect of RAASi on the primary outcome, using multiple imputation techniques for the ITT analysis."

• The investigators go on and on but never make clear what statistical method was applied to the primary outcome. They state that "A binary response variable was created to reflect the composite clinical outcome among participants." But they never make clear what test they used nor do they explain why they conducted a per protocol analysis first followed by an ITT analysis-or so it appears. I also do not understand why the primary outcomes is treated as a binary variable when they state that the primary outcome is both achieving target RAASi dose and a serum potassium below 5.6 mmol/L. Binary usually means to mutually exclusive events. Please explain.

• Further, the statistical analytical method, they state they state that they employed mixed effects models for all biochemical parameters but they do not explain what variables were included in these models.

• There is some imbalance in the number of participants randomized to the blinded intervention arms, 53 vs 59. This is not a small difference in a study of just over 100 participants. What is the reason for this imbalance?

• I do not understand why the investigators defined study dropout as “absence at a scheduled follow up visit”. Does this mean any visit? Would the participant not be allowed to be considered to be still in this study if they drop out by this definition?

• There is a significant bias introduced in the analysis as 53 SZC and only 43 placebo participants were analyzed for the primary outcome due to lower number randomized and higher number of discontinuations in the placebo group (See CONSORT figure 1).
• In terms of secondary outcome of achieving target RAASi, the difference barely made statistical significance at the 5% level by ITT.

• The paragraph about discrepancies in inference on page 10 is not understandable. It is rather circuitous and confusing. And ends with a phrase I have never seen in a scientific manuscript: “We have found that, early failures hyperkalaemia (≥5.6mmol/L) for the two patients in the SCZ group would drift away the statistical significance at 0.05 level between the two intervention groups.” What does “drift away from statistical significance mean? It has no meaning to me, please explain.

• The investigators state that a small number of admissions occurred. I assume they mean hospitalizations but it is not clear. And if these are hospitalizations it would help to know whether or not these were hospitalizations for heart failure and if the 2 participants in the SZC who were admitted were admitted for heart failure. And if they were heart failure hospitalizations it would be of interest to know dose of SZC they were on at the time of admission.
• In the discussion the investigators claim the strength of their study is that it is “real-world” population. I challenge this. What is different from other randomized double-blind placebo controlled trials in HF and CKD populations. This was not a pragmatic trial. Each participant was approached individually and strict inclusion and exclusion criteria applied. I think it is misleading to suggest this is a “real-world” trial. Many studies in people with HF and CKD include older patient populations.
• The manuscript does not support the specific statement made in the conclusion. Specifically, the investigators do not quantify “rapid uptitration” as the paper provides no data on the rate of uptitration of RAASi. There is no data nor mention of rate of uptitration in the entire manuscript.

Minor
• The investigators should comment on the fact that only 25% of the study population were female. A 3:1 ratio of male:female for HFrEF is not what is generally observed in the broader population.
• It would be helpful to list the distribution of participants by stage of CKD in Table 1.
• There are no figure legends for the plethora of plots starting on page 28 of the manuscript.

Reviewer #2

(Remarks to the Author)
This is an interesting study. I have the following comments.

Methods
p.5, line 104. It stated that “… by the study pharmacist according to the randomisation schedule”. Please provide more details about the randomisation schedule.
For the mixed effects logistic regression models, please indicate clearly about the random effects structure, the fixed effects, and the estimation methods used in the models. What’re the covariates included in the GEEs models?
It’s not clear the covariates used in the Cox-regression models.
P.7, lines 155-158. It looks that both Poisson and negative binomial regression models were used for analysing count data in the analysis. Suggest using the one that provides more reliable results.
P.7, line 169. “Mixed effects models were also conducted for all biochemical markers such Potassium, eGFR,…”. Suggest specifying the models, such as linear mixed effects models if that’s the case.

Results
P.10, line 241-242. “0.02 mmol/L (95% CI 0.00–0.05)”. Suggest changing 0.00 to 0.002 as shown in the table.
P.10, line 245. “95% CI 0.0001–0.06”. 0.0001 seems to be 0.001. Please check.
Table 1. Please add the number of participants in sodium zirconium group and keep variables (mean and SD) in the same format within the table.
Table 2. Note that the primary outcomes were analysed using both per-protocol and intention-to-treat (ITT) approaches. This’s a superiority study to compare differences between two randomised groups. The ITT principle is preferred approach for analysing the data. Caution should be made when interpreting the results based on “per protocol” analysis. In addition, the meaning of “Reached Potassium 5.6” was not clear.
Table 3. All abbreviations need to be clearly defined in the table.
Table 4. Please indicate the covariates used the models.
Table 5. In the table, different versions of the log-rank test were presented. It would be better to select the most appropriate ones.

Reviewer #3

(Remarks to the Author)
Banerjee et al. report data from their double blind placebo controlled to test whether treatment with a potassium binder (SZC) resulted in lower rates of hyperkalemia and better uptitration of HF medication in patients with impaired renal function.
Overall this is an interesting topic, although not completely novel. Several trials have been executed to test this hypothesis (i.e. uptitration RAASi in patients with HF): including PEARL, DIAMOND and REALIZE-K, only the latter one is mentioned in the manuscript. Furthermore, there are concerns that the sodium load in ZSC may deteriorate HF (as has been observed in the the REALIZE-K trial.
In the current trial the authors find that SZC leads to more uptitration and less hyperK. The analyses, tables and figure are overwhelming and not very clear. Also the combination of reporting of ITT and per protocol in the same paragraphs are confusing. The paper would benefit from a clearer presentation, including less tables and a focus on the main outcome. The drop-out is relatively high. I miss a paragraph on safety.

Specific comments:
The authors mention that a powercalculation was published previously, however it is crucial to include a summary in the main paper.

How did the authors deal with the two components of primary endpoint: target dose RAASi and K<5.6? Was this prespecified? Do the authors have a statistical analysis plan?

How was the RAASi uptitration protocol defined?

It is unclear which dose of RASi / MRA patients were using at the moment of randomization. This should be in the baseline table. It is also relevant to report this in the outcome section: i.e. dose ACEi achieved in both groups; MRA dose achieved; were ARNI's prescribed?

From the methods section it is unclear what the author mean with “Final RAASi dosing was determined using a tailored, semi-automated Stata script developed and implemented by the study statistician in collaboration with the clinical lead”. What kind of script are the authors refering to?

How was target dose defined? Is there evidence that 25mg spironolactone is superior to 50mg?

The difference between previous trials with potassium binders to enable uptitration of HF medication is that patients with eGFR<30 were allowed to participate. As such it would be important to better specify this subgroup.

Results section:
Please include linear troponin and NTproBNP data (line 178); log scale makes them uninterpretable to a clinician.

It is confusing to report in the same paragraph the ITT and per-protocol analysis. As a sensitivity analysis per-protocol can be mentioned at the end of the results section.

Drop out was double for the SZC group (10% vs 19%). What was the reason for drop out?

The authors report (line 187) 24 of 43 patients, but in the itt population this should be 53. What happened to the other 10 patients? Again, the primary outcome should be ITT.

The tables are difficult to read. Why not report % diabetes, %hypertension. Please report only IQR is non-normally distributed. Providing both mean and IQR makes it overly complicated to read.

Figure 1; difficult to read: what does potassium 5.6 Yes mean? (below 5.6? above?)

Version 1:

Reviewer comments:

Reviewer #1

(Remarks to the Author)
The manuscript is substantially improved and the authors responses are satisfactory.

Reviewer #2

(Remarks to the Author)
The authors have responded well to my questions. Noted that the same methods for analysing biochemical markers (potassium, eGFR, sodium, troponin, NT-proBNP, and ACR) were described repeatedly in the statistical methods on page 8 and 11.

Reviewer #3

(Remarks to the Author)

The authors responded to all my comments. I have no additional comments, but I think the manuscript would benefit from removing the per-protocol analysis/results to the supplements.

Open Access This Peer Review File is licensed under a Creative Commons Attribution 4.0 International License, which permits use, sharing, adaptation, distribution and reproduction in any medium or format, as long as you give appropriate credit to the original author(s) and the source, provide a link to the Creative Commons license, and indicate if changes were made.
In cases where reviewers are anonymous, credit should be given to 'Anonymous Referee' and the source.
The images or other third party material in this Peer Review File are included in the article's Creative Commons license, unless indicated otherwise in a credit line to the material. If material is not included in the article's Creative Commons license and your intended use is not permitted by statutory regulation or exceeds the permitted use, you will need to obtain permission directly from the copyright holder.
To view a copy of this license, visit https://creativecommons.org/licenses/by/4.0/

Response to Reviewers

Nature Communications Manuscript#: NCOMMS-25-97999-T

We sincerely thank the reviewers for the careful evaluation of our manuscript and for the thoughtful, detailed, and constructive feedback. We greatly appreciate the time and expertise devoted to reviewing our work, and we believe that these comments have substantially improved the quality and clarity of the manuscript. We are grateful for the opportunity to revise our submission and to address the points raised.

We particularly appreciate the reviewers' insights into the challenges of managing this population of chronically ill and highly vulnerable patients, as well as their emphasis on the potential applicability of our approach in routine clinical practice.

We also apologise for the omission of the figure legends and for the lack of certain important details regarding patient management in the original submission. These issues have now been carefully addressed in the revised manuscript, mainly in the methods section, which has been restructured with five additional supplementary tables and one supplementary figure.

Below, we provide a detailed point-by-point response to each comment. For ease of reading, the reviewers' comments are presented in black, followed by our responses in blue. Changes made to the manuscript are indicated in blue italics within the responses.

Reviewer #1 (Remarks to the Author):

Summary
The investigators conducted a double-blind, placebo-controlled, randomised trial to determine whether sodium zirconium cyclosilicate (SZC) enables renin-angiotensin-aldosteone inhibitors (RAASi) up-titration in patients with stage 2-5 chronic kidney disease (CKD) and reduced ejection fraction heart failure (HFrEF) and a serum potassium of 5.0–5.5mmol/L over 14 weeks. 112 patients were randomized to SZC (n=53) or placebo (n=59). Patients in the SZC group were 3 times more likely to reach the target dose without reaching potassium 5.6mmol/L (OR= 3.16; 95% CI 1.35–7.39; P=0.008), than in the placebo group - intention-to-treat analysis (ITT). Hyperkalaemia occurred in 28% of SZC patients compared to 49% with placebo (P=0.036 ITT analysis (HR=0.48; 95%CI 0.25–0.91). The trial did not find significant differences between two groups in quality of life, biomarkers or adverse events. SZC appears to safely support rapid RAASi optimisation in HFrEF patients with CKD.

Critique
Achieving guideline directed medical therapy in patients with HFrEF or advanced CKD or both is important for reducing risk for kidney failure and cardiovascular death. However, as the investigators point out hyperkalemia is a major obstacle to achieving GDMT in these patient populations because of their underlying diseases and the need for RAASi as part of the GDMT. Many real world studies observational studies have used administrative data to determine whether potassium binders enable use of RAASi and the findings suggest that their use is effective. However, few studies have carefully examined the use of potassium binders, including SZC, to improve clinician's ability to optimize GDMT in high risk populations such as those with stages 3-5 CKD and documented HFrEF. The investigators are to be commended for conducting a randomized double-blind placebo-controlled trial to address this issue. While the findings support their conclusion the trial was 14 weeks in duration and could not answer the more pressing question of whether enabling use of RAASi makes a difference in clinical outcomes including kidney failure, heart failure and mortality. And, unfortunately, there are numerous concerns with the study design and conduct that raise serious concerns about the conclusion as detailed below.

Strengths
• The patient population under study is a strength. This is a complicated and high risk group of patients that need to be studied carefully as the investigators recognize
• The double-blind placebo controlled design is another strength
• The use of SZC as a test agent is another strength as it has proven efficacy to lower potassium in patients with heart failure, kidney disease, or both and is well tolerated by patients.
• Evaluation of patient reported outcomes is a strength

We would like to thank the reviewer for thoughtfully highlighting the strengths of our study, which reflect the depth and thoroughness of our research and the need for the RCT in the high-risk population. Our responses to each point are provided below in detail.

Weaknesses
Major

• The major weakness of this manuscript is that it provide no guidance for clinicians on how to uptitrate RAASi. While I believe that they have provided sufficiently robust data about the enabling effect of SZC on uptitration of RAAS (with some reservation given the discordant number of participants completing the trial in the SZC vs placebo) group, they do not explain how the uptitration was done let alone the rate at which uptitration was successfully achieved. So, as a clinician I can believe the data but it is no help to me in managing the patient. What the investigators have shown is a proof of concept but it is of little or no clinical value to the broader community of clinicians caring for these very chronically ill and highly vulnerable patients.

We are grateful to the reviewer for emphasising the importance of detailing our experience with RAAS inhibitor up-titration in this cohort of chronically ill and highly vulnerable patients.

At each study visit, two weeks apart, the treating physician—blinded to treatment allocation—was instructed to up-titrate RAAS inhibitors according to the study protocol, progressing from the starting dose to the half maximal dose and subsequently to the maximum dose at two-week intervals, provided it was safe to do so on clinical and biochemical evaluation. Up-titration was undertaken provided that patients were not hypotensive (systolic blood pressure <90 mmHg), had no symptomatic or clinically significant postural drop in systolic blood pressure (≤20 mmHg), were not hyperkalaemic, and had not experienced significant adverse effects at the previous dose.

The starting, half maximal, and maximum doses of ARNI, ACE inhibitors, ARBs, and MRAs are detailed in Table S2. At each visit, potassium levels were assessed using both an urgent point-of-care test and a standard laboratory measurement.

Table S2: The minimum, half-maximal, and maximum dose of ACEi/ARB/ARNI and MRA

	**Minimum dose**	**Half maximal dose**	**Maximum dose**
**ACEi/ARB/ARNI (one of the following)**			
Ramipril	2.5 mg OD	5 mg OD	10 mg OD
Perindopril arginine	2.5 mg OD	5 mg OD	10 mg OD
Enalapril	2.5 mg OD	5 mg OD	10/20/40 mg OD
Lisinopril	2.5 mg OD	5 / 10 mg OD	20 / 40 mg OD
Perindopril erbumine	2 mg OD	4 mg OD	8 / 16 mg OD
Sacubitril/valsartan	24 / 26 mg BD	49 / 51 mg BD	97 / 103 mg BD
Losartan	25 mg OD	50 mg OD	100 mg OD
Candesartan	4 mg OD	8 / 16 mg OD	32 mg OD
Irbesartan	75 mg OD	150 mg OD	300 mg OD
Telmisartan	20 mg OD	40 mg OD	80 mg OD
Olmesartan	10 mg OD	20 mg OD	40 mg OD
Valsartan	40 mg BD	80 mg BD	160 mg BD
**MRA (one of the following)**			
Spironolactone	12.5 mg OD	25 mg OD	50 mg OD
Eplerenone	12.5 mg OD	25 mg OD	50 mg OD

Legend: The study clinician was instructed to increase from starting, to half-maximal, and maximum dose provided it was safe to do so. See table S3 for more information. BD – twice

a day; OD – once a day; ARNI – Angiotensin receptor neprilysin inhibitor; MRA – mineralocorticoid receptor antagonist.

The dose of placebo or sodium zirconium cyclosilicate was adjusted according to the study protocol. Specifically, treatment was withheld when serum potassium was <3.5 mmol/L and increased when potassium exceeded 5.0 mmol/L, as detailed in Table S1.

Patients with a potassium level >6.0 mmol/L on point-of-care testing, confirmed by urgent laboratory measurement, were immediately referred from the clinical research facility to the on-site emergency department for management of hyperkalaemia.

Table S1: Instructions for changing the dose of IMP at every 2-weekly study visits

SZC or Placebo dose at start of visit	Serum potassium end of visit	Dose change at the end of visit
If on 10 g daily	> 5.0 mmol/L (but ≤ 6.0 mmol/L)	Continue 10 g daily
	3.5–5.0 mmol/L	Change to 5 g daily
	< 3.5 mmol/L	Stop
	> 6.0 mmol/L	Stop and initiate rescue
If on 5 g daily	> 5.0 mmol/L	Change to 10 g daily
	3.5–5.0 mmol/L	Continue 5 g daily
	< 3.5 mmol/L	Stop
If on 0 g daily (temporary stop)	≤ 5.0 mmol/L	Continue with no IMP
	> 5.0 mmol/L	Initiate 5 g daily

Legend: The IMP (SZC or placebo) was changed depending on serum potassium and previous dose of IMP. IMP – investigational medicinal product; SZC – sodium zirconium cyclosilicate

Figure S1: RAASi dose in each visit during the study

Figure S1 A and B: The dose of ACEi/ARB/ARNI (A); The dose of MRA in 2-weekly study visits (B) : The bars indicate the number of participants on none, minimum dose, half maximal, and maximum dose of RASi and MRA in visits 1-7 two weeks apart stratified by SZC and Placebo arms. *RASi – renin-angiotensin system inhibitor; MRA – mineralocorticoid receptor antagonist; SZC – sodium zirconium cyclosilicate*

A

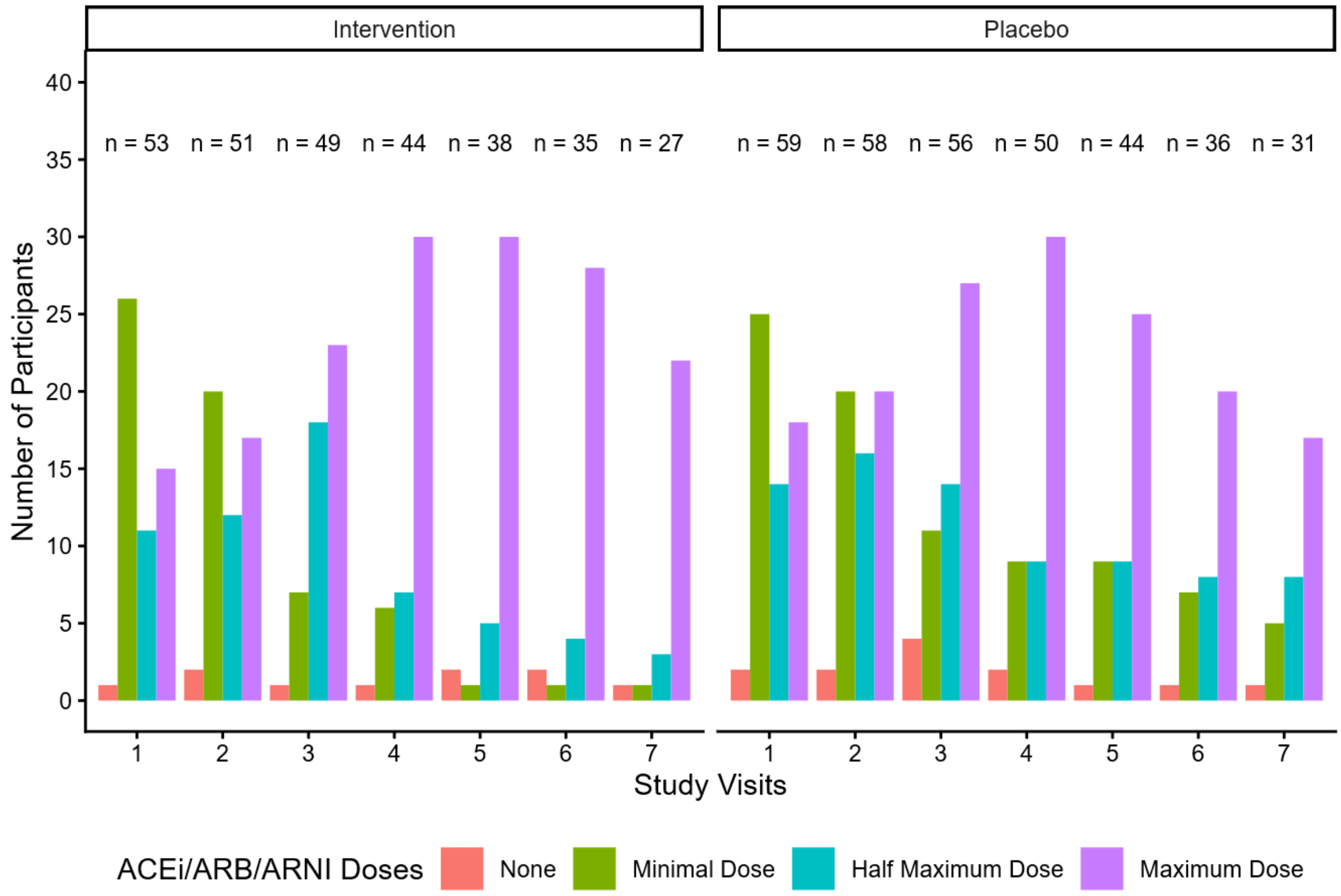

ACEi/ARB/ARNI Escalation

B

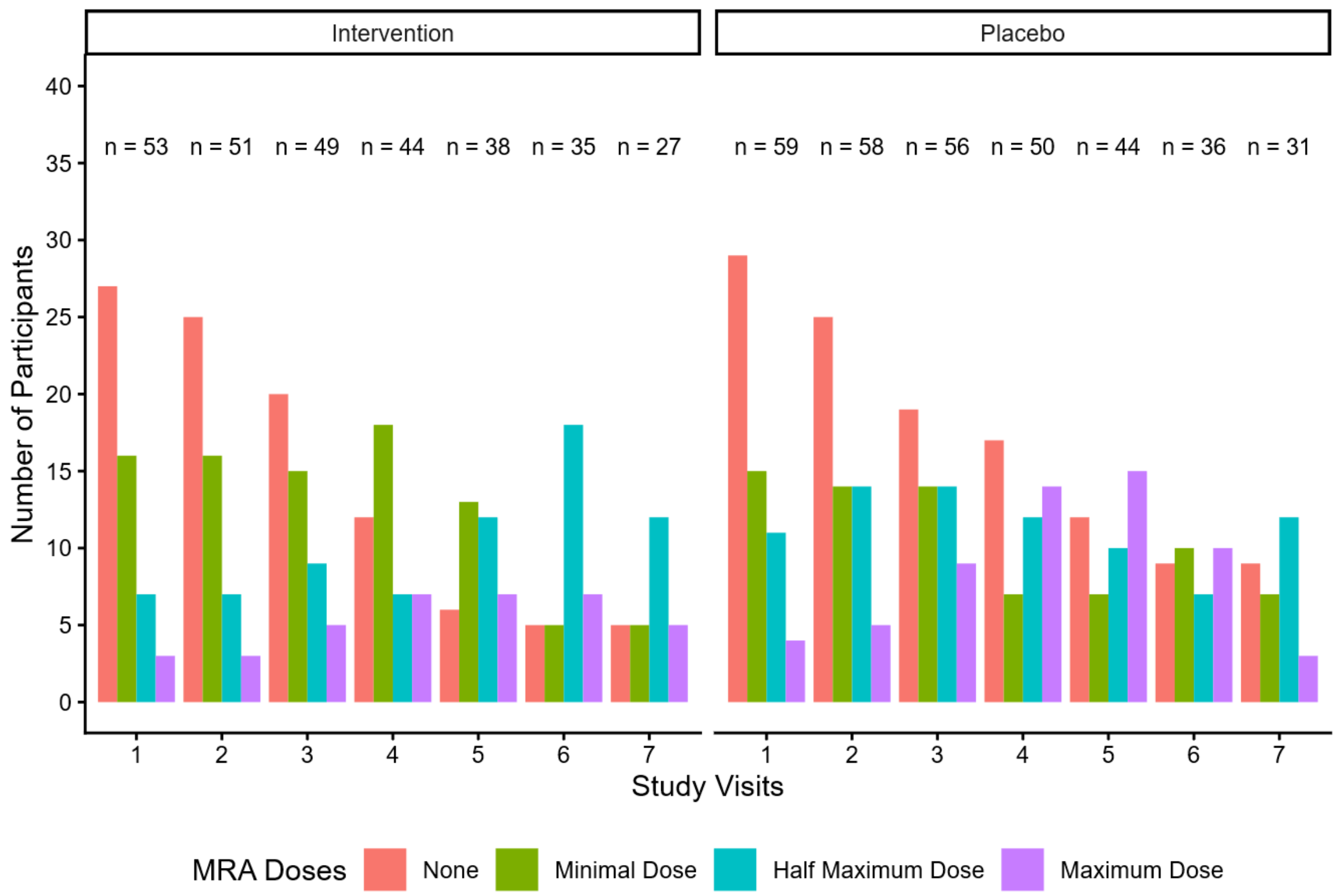

MRA Escalation

We added the following to the methods section under intervention subsection

"Patients with serum potassium of 3.5 – 5.0 were treated with 5g daily of IMP, while treatment was withheld when serum potassium was <3.5 mmol/L and increased to 10g daily when potassium exceeded 5.0 mmol/L, as detailed in Table S1. Patients with a potassium level >6.0 mmol/L on point-of-care testing, confirmed by urgent laboratory measurement, were immediately referred from the clinical research facility to the on-site emergency department for management of hyperkalaemia.

At each study visit, two weeks apart, the treating physician—blinded to treatment allocation—was instructed to up-titrate RAAS inhibitors according to the study protocol, progressing from the starting dose to the half-maximal and subsequently to the maximum dose at two-week intervals, provided it was safe to do so on clinical and biochemical evaluation (Table S2). Up-titration was undertaken provided the patients were not hypotensive (systolic blood pressure <90 mmHg), had no symptomatic or clinically significant postural drop in systolic blood pressure (≤20 mmHg), were not hyperkalaemic, and had not experienced significant adverse effects at the previous dose. Table S3 outlines the recommended dose escalation for patients not receiving any RAASi inhibitors at baseline and Table S5 shows the specific RAASi agents used.

The primary objective of this study was to compare the proportion of patients in the SZC group versus the placebo group who achieved the target RAASi dose while maintaining a serum potassium concentration below 5.6 mmol/L."

• The conclusion should be amended or the investigators should illustrate rapid titration, that is how quickly or slowly the RAASi was titrated. The only thing I can find regarding "rapid" uptitration is that they protocol called for titrating on a 2week basis and this was done in a 14 week trial. But the investigators cite no literature that defines robustly what "rapid" uptitration means. To put this in the title of the paper and in the conclusion with no scientific evidence to support it, seems unconvincing to me.

We are grateful to the reviewer for highlighting the need to clarify what is meant by rapid up-titration of RAAS inhibitors in this multimorbid and clinically vulnerable population requiring close monitoring.

Our protocol was similar to the approach used in the Safety, Tolerability and Efficacy of up-titration of guideline directed medical therapies for acute heart failure (Strong-HF): a multinational open label RCT (Mebazza et al Lancet 2022 400 1938) study, which evaluated rapid up-titration of guideline-directed medical therapy, including RAAS inhibitors, in patients with acute heart failure.[1] In that study, treatment was escalated from half to full dose over two weeks, with safety assessments at weeks 1, 2, 3, and 6. Notably, baseline use of ACEi/ARB/ARNI and MRA was high (64% and 94%, respectively), and overall kidney function was better than in our cohort (baseline eGFR in intervention arms 61.92 (19.92) versus 34.72 (11.96) ml/min/1.73m2 in our study) study).

In our study, RAAS inhibitor doses were increased in a stepwise manner from the starting dose to the half-maximal dose and subsequently to the maximum dose at two-week intervals, provided patients did not develop hypotension, hyperkalaemia, or treatment intolerance. Consistent with the Strong-HF protocol, safety monitoring included clinical review and measurement of electrolytes, BNP and creatinine at each visit.

Importantly, despite the greater clinical complexity and more advanced renal impairment in our population, this up-titration strategy was both feasible and well tolerated. There was no evidence to support a shift in the Quality of life between treatment arms in our study. In Strong-HF, quality of life improved at 90 days in the high-intensity treatment group.

We have added in the Discussion section the paragraphs below:

"Our protocol was similar to the approach used in the Safety, Tolerability and Efficacy of up-titration of guideline directed medical therapies for acute heart failure (Strong-HF): a multinational open label RCT study, which evaluated rapid up-titration of guideline-directed medical therapy, including RAAS inhibitors, in patients with acute heart failure; treatment was escalated from half to full dose over two weeks, with safety assessments at weeks 1, 2, 3, and 6.[1]

Notably, baseline use of ACEi/ARB/ARNI and MRA was high (64% and 94%, respectively), and overall kidney function was better than in our cohort (baseline eGFR in intervention arms 61.92 (19.92) versus 34.72 (11.96) ml/min/1.73m2 in our study).

In our study, RAAS inhibitor doses were increased in a stepwise manner from the starting dose to the half-maximal dose and subsequently to the maximum dose at two-week intervals, provided patients did not develop hypotension, hyperkalaemia, or treatment intolerance. Consistent with the Strong-HF protocol, safety monitoring included clinical review and measurement of electrolytes, BNP and creatinine at each visit.

Importantly, despite the greater clinical complexity and more advanced renal impairment in our population, this up-titration strategy was both feasible and well tolerated. There was no evidence to suggest a shift in the quality of life between treatment arms in our study (See Table 7). *In Strong-HF, quality of life improved at 90 days in the high-intensity treatment group."*

• In review of the LIFT protocol, it is not clear that all participants had an echocardiographic or cardiac MRI to confirm HFrEF. The inclusion criteria from LIFT states that Heart failure was diagnosed on clinical grounds or echo confirmed. It seems that they only required an echo for inclusion if the potential participant had atrial fibrillation. How do the investigators know that a patient with :clinical" diagnosis of heart failure has HFrEF vs HFpEF?

We thank the reviewer for observing the missing information regarding cardiac imaging in the manuscript. All included patients underwent an echocardiogram or an MRI scan to diagnose HFrEF with EF ≤40%.

We have added the sentence in the Methods section under Patients subsection:

"All included patients underwent an echocardiogram or an MRI scan to diagnose HFrEF with EF ≤40%"

• The manuscript should include the definition of the target dose of RAASi and not just refer to the LIFT trial protocol. The LIFT publication does not explicitly state how the target dose of RAASi was achieved. The study flow char in Fig 1 of the paper only describes use of spironolactone and ramipril but no other RAASi that participants are permitted to use.

This is important for three reasons:

1) the primary outcome of the study is the proportion of patients in SZC vs placebo who achieved the target RAASi dose while maintaining a serum potassium below 5.6 mmol/L and

2) to understand how the target RAASi and

3) to understand whether multiple algorithms were used, for example tailored to the patient depending on the severity of HFrEF and/or CKD. None of this is specified in the Methods section of this manuscript.?

We thank the reviewer for requesting further details on RAAS inhibitor use in the trial.

The dose up-titration protocol for trial medications was standardised across all participants. Table S2 provides details of the starting dose, half-maximal dose, and maximal dose for each medication. For example, the starting, half-maximal, and maximal doses of eplerenone and spironolactone were 12.5 mg, 25 mg, and 50 mg, respectively. For sacubitril/valsartan, the corresponding doses were 24/26 mg twice daily, 49/51 mg twice daily, and 97/103 mg twice daily. ARNI was used in 73% of participants.

We have also added Supplementary Table S3, which outlines the recommended dose escalation for patients not receiving any RAAS inhibitors at baseline, and Supplementary Table S5, which summarises the agents used at the start and at the end of the study.

Table S3: Guidance on dose escalation of RASi (ACEi/ARB/ARNI) and MRA in 2-weekly study visits

	Ideal Doses*						
Visits	Visit 1	Visit 2	Visit 3	Visit 4	Visit 5	Visit 6	Visit 7
RASi (ACEi/ARB/ARNI) (one of the following)							
Sacubitril/Valsartan *(Twice a day)*	0mg	24/26mg	24/26 mg	49/51 mg	49/51 mg	97/103 mg	97/103 mg
Ramipril *(once a day)*	0mg	2.5mg	2.5 mg	5mg	5mg	10 mg	10 mg
Candesartan *(once a day)*	0mg	4 mg	4 mg	8 mg	16 mg	32 mg	32 mg
		Minimum dose		Half maximal dose		Maximum dose	
MRA (one of the following)							
Eplerenone (Once a day)	0mg	0mg	12.5mg	12.5mg	25mg	25mg	50 mg
Spironolactone (Once a day)	0mg	0mg	12.5mg	12.5mg	25 mg	25 mg	50 mg
			Minimum dose		Half maximal dose		Maximum dose

Legend: Table showing how dose could have been escalated in a patient starting with no RAASi where maximum dose can be achieved over 7 study visits. *RASi – renin-angiotensin system inhibitor; MRA – mineralocorticoid receptor antagonist*

Table S5: Specific agents used during the study in all patients

Agent	At baseline	At study end
Sacubitril/Valsartan	83	82
Ramipril	16	13

Candesartan	8	7
Losartan	1	1
Irbesartan		1
Eplerenone	37	58
Spironolactone	19	27

Legend: Table shows the specific RASi and MRA agents used within the trial at the start of study and study end. *RASi – renin-angiotensin system inhibitor; MRA – mineralocorticoid receptor antagonist*

We have added to the Methods section the sentences below:

"Table S3 outlines the recommended dose escalation for patients not receiving any RAASi inhibitors at baseline and Table S5 shows the specific RAASi agents used."

May we add that we accept that the terminology such as "using an algorithm" was rather confusing. That was in reference to a part of the analytical methodology which calculated the allocated doses and was post data collection. Medication allocation decision was determined by participant's current RAASi dose, serum potassium, blood pressure, medicine tolerance and was exclusively clinicians' responsibility.

• The Methods section does not explain where the participants were recruited from. Were participants recruited from cardiology, nephrology, general medicine clinics? Were hospitalized patients eligible for the study?

We thank the reviewer for the comment. All patients were ambulatory at the start of the study. The patients were recruited from cardiology, nephrology and cardiorenal clinics.

We have added the following sentence to the Methods section, under Patients subsection:

"*All patients were recruited from the cardiology, nephrology and cardiorenal clinics and were ambulatory at baseline*"

• Further, the investigators allude to a semi-automated Stat script to tailor final RAASi dosing but they do not provide the specifics. This is critically important to analyzing the results of the study and particularly important if the findings are to be translated into clinical practice. Otherwise, how can a clinician use the data from this manuscript to help their patients?

We thank the reviewer for highlighting this important point and apologise for our phrasing which created confusion. We feel that we need now to clarify that treatment decisions during the trial were made by clinicians in accordance with the study protocol, whereas the Stata routine has been created and applied with the sole purpose of dose calculation – after data collection to consistently map observed RAASi doses onto the predefined dose categories (Table S2) for analysis. This distinction reflects the role of the script as an analytical implementation of the protocol rather than a clinical decision-making tool which was not our intended communication.

RAASi up titration was done as mentioned in response above using the text in the methods section (intervention subsection) and table S2 and S3. The actual doses are in figure S1 A&B.

We have changed the Methods (Intervention subsection) which reads now as:

“The RAASi optimisation treatment period ranged from 2 to 12 weeks, depending on the patient’s baseline RAASi dose, with follow-up extending from 4 to 14 weeks. Data were collected at each scheduled study visit. The investigational medicinal product (IMP) was self-administered at home in 5- or 10-gram sachets, in accordance with a standardised protocol[2,3]. At each visit, potassium levels were assessed using both an urgent point-of-care test and a standard laboratory measurement. Patients with serum potassium of 3.5 – 5.0 were treated with 5g daily of IMP, while treatment was withheld when serum potassium was <3.5 mmol/L and increased to 10g daily when potassium exceeded 5.0 mmol/L, as detailed in Table S1. Patients with a potassium level >6.0 mmol/L on point-of-care testing, confirmed by urgent laboratory measurement, were immediately referred from the clinical research facility to the on-site emergency department for management of hyperkalaemia.

At each study visit, two weeks apart, the treating physician—blinded to treatment allocation—was instructed to up-titrate RAAS inhibitors according to the study protocol, progressing from the starting dose to the half-maximal dose and subsequently to the maximum dose at two-week intervals, provided it was safe to do so on clinical and biochemical evaluation (Table S2). Up-titration was undertaken provided the patients were not hypotensive (systolic blood pressure <90 mmHg), had no symptomatic or clinically significant postural drop in systolic blood pressure (≤20 mmHg), were not hyperkalaemic, and had not experienced significant adverse effects at the previous dose. Table S3 outlines the recommended dose escalation for patients not receiving any RAASi inhibitors at baseline and Table S5 shows the specific RAASi agents used.

The primary objective of this study was to compare the proportion of patients in the SZC group versus the placebo group who achieved the target RAASi dose while maintaining a serum potassium concentration below 5.6 mmol/L.”

• They state that separate binary outcomes were also specified for RAASI dose achievement and occurrence of hyperkalemia including a separate one for severe cases, but they do not define “severe cases”. What does severe mean in this context?

Thank you for drawing attention to this point. We had previously specified these definitions later in the manuscript, but we agree that they should be stated more clearly earlier in the text. Accordingly, we have now clarified the definitions of hyperkalaemia and severe hyperkalaemia at the point of their first introduction. The revised text under Outcomes’ definitions reads:

“The primary endpoint was defined as a composite binary outcome, with success defined as achieving both the target RAASi dose and maintaining a serum potassium concentration less than 5.6 mmol/L throughout the trial. Failure was defined as either not achieving the target RAASi dose or achieving it in the presence of a serum potassium concentration reaching 5.6 mmol/L or higher.”

We have been also more specific in our univariate binary outcomes definitions under Outcomes’ Definitions, which reads now as below:

“Three other secondary binary outcomes were defined for (i) achievement of the target RAASi dose or not, (ii) first occurrence of hyperkalaemia (serum potassium ≥5.6 mmol/L) or not, and (iii) first occurrence of severe hyperkalaemia (serum potassium ≥6.0 mmol/L) or not – the later at least once during trial follow-up. ”

• The manuscript notes that patients provided informed consent but they do not indicate whether or not the protocol was approved by an institutional review board or ethics

committee.

Thank you for noting this important omission. The study protocol includes the Research Ethics Committee approval and approval from the Medicines and Healthcare Regulatory Authority. We have now added the following statement to the manuscript at the end of the Methods section to ensure clarity:

"The study received ethical approval from the Cambridge NHS Research Ethics Committee 20/EE/0227 and the Medicines and Healthcare Regulatory Authority(CTA 12853/0009/001-0001)"

• The manuscript does not state whether a data monitoring committee (DMC) was involved in the trial and if so what their role was in monitoring the conduct of the trial. They state that the study was not unblinded until it was completed suggesting that no one including a statistician was involved in viewing the data. While I understand this is not a hard clinical outcome trial many institutions require a DMC for a multisite study especially in such a high risk population. Even many single center studies employ a DMC.

We thank the reviewer for raising this important point regarding trial oversight. The study was conducted as a double-blind trial, with treatment allocation concealed from all investigators, including the study statistician, until completion of data collection and database lock. In accordance with the trial protocol, no early conclusions were drawn prior to database lock.

As pre-specified in the protocol, interim analyses were planned to assess feasibility and tolerability after approximately 10%, 50%, and 75% of participants had completed the study. These reviews were conducted using blinded data and were focused on trial conduct, safety signals, and data completeness, without unblinding treatment allocation.

Our formal independent Data Monitoring Committee (DMC) headed by two independent nephrologist (Dr Richard Hull- chair and Dr Edward Stern from St George's Hospital) and one independent cardiologist (Dr Robin Ray also from St George's Hospital), oversighted the process through multiple mechanisms. The study management team Data Monitoring Committee met every three months during the trial to review recruitment, protocol adherence, safety considerations, and data quality, in line with the protocol. In addition, data were collected via secure electronic case report forms (eCRFs) and were accessible only to authorised study personnel. The study also permitted monitoring, audit, and inspection by authorised representatives of the Sponsor from the host institution St George's Joint Research and Enterprise service, and regulatory authorities, in accordance with Good Clinical Practice and applicable data protection regulations. The sponsor reported all adverse events to the UK Government Medicine Health Regulatory Agency and funder.

We have also had a Trial Management Steering Committee chaired by Professor Indranil Dasgupta from University Hospital Birmingham together with Professor Guiseppe Rosano from St Georges Hospital which also met four time during the trial who monitored trial progress, recruitment, reports from DMC and other safety considerations. The trial management committee met every 7 to 30 days to discuss recruitment and other issues regarding running of the trial and implement the changes suggested by TSC and DMC if necessary.

Given the single-centre design, the established safety profile of the investigational product, and the protocol-defined monitoring and governance procedures, this approach as considered appropriate. We have now clarified these aspects in the manuscript to improve transparency.

We have added the following in the methods section in Ethics approval and Trial Monitoring

"Trial oversight was ensured through a predefined governance structure by the study sponsor St George's Joint Research and Enterprise service. The study was conducted as a double-blind trial, with treatment allocation concealed from all investigators, including the study statistician, until completion of data collection and database lock. Interim analyses were pre-specified in the protocol and undertaken after approximately 10%, 50%, and 75% of participants had completed follow-up. These analyses were performed on blinded data and focused on feasibility, tolerability, safety signals, and data completeness, without unblinding treatment allocation.

An independent Data Monitoring Committee (DMC), comprising two nephrologists and one cardiologist, provided oversight throughout the trial. A Trial Management Committee, chaired by an independent clinician and including senior investigators, met periodically to review trial conduct. In addition, the study management team met quarterly to assess recruitment, protocol adherence, safety, and data quality. Data were collected using secure electronic case report forms and were accessible only to authorised personnel. The study was conducted in accordance with Good Clinical Practice and was subject to monitoring, audit, and inspection by authorised representatives of the sponsor, host institution, and regulatory authorities.

The Data Monitoring Committee met every three months during the trial to review recruitment, protocol adherence, safety considerations, and data quality, in line with the protocol. The sponsor reported all adverse events to the UK Government Medicine Health Regulatory Agency and funder. The Trial Steering Committee met every 6-monthly to monitor the progression of the trial, patient safety, research governance, protocol, and report from the DMC. The trial management committee met every 7 to 30 days to discuss recruitment and other issues regarding running of the trial and implement the changes suggested by TSC and DMC if necessary."

• I find the statistical methods very confusing. The investigators do not state clearly what statistical method was used for the primary outcome. Instead, they make general statements about analyses, for example: "For binary and categorical univariate outcomes, treatment effects were first evaluated in the per-protocol (PP) analysis using χ^2 or Fisher's exact tests. In the intention-to-treat (ITT) analysis we used the permutation-based versions of these tests, which preserve the randomization structure of the data and provide exact inference without the need for distributional assumptions. We have also estimated the odds ratios for the effect of RAASi on the primary outcome, using multiple imputation techniques for the ITT analysis."

Thank you very much for this observation – the answers to this question are combined with those to the question below.

• The investigators go on and on but never make clear what statistical method was applied to the primary outcome. They state that "A binary response variable was created to reflect the composite clinical outcome among participants." But they never make clear what test

they used nor do they explain why they conducted a per protocol analysis first followed by an ITT analysis-or so it appears. I also do not understand why the primary outcomes is treated as a binary variable when they state that the primary outcome is both achieving target RAASi dose and a serum potassium below 5.6 mmol/L. Binary usually means to mutually exclusive events. Please explain.

We thank the reviewer for these helpful comments. We recognise that the definition of the primary binary outcome was not presented with sufficient clarity in the original manuscript.

To address this, we have revised and restructured the Methods section.

The newly added *Outcomes' Definitions* subsection of the Methods contains these definitions which we hope enhanced clarity:

"The primary endpoint was defined as a composite binary outcome, with success defined as achieving both the target RAASi dose and maintaining a serum potassium concentration less than 5.6 mmol/L throughout the trial. Failure was defined as either not achieving the target RAASi dose or achieving it in the presence of a serum potassium concentration reaching 5.6 mmol/L or higher.

A refined four-level categorical response was defined by jointly classifying the target dose achievement (YES or NO) and hyperkalaemia status (serum potassium ≥5.6 mmol/L or <5.6 mmol/L).

Three other different secondary binary outcomes were defined for (i) achievement of the target RAASi dose or not, (ii) first occurrence of hyperkalaemia (serum potassium ≥5.6 mmol/L) or not, and (iii) first occurrence of severe hyperkalaemia (serum potassium ≥6.0 mmol/L) or not."

The subsequent longitudinal outcomes definitions remain unchanged in this section.

We have been more specific in the *Statistical Analytical Methods* subsection on the specific test that we have used to analyse the primary outcomes, its refined counterpart and the other binary outcomes defined in the *Outcomes' Definitions* section:

For binary and categorical univariate outcomes (the primary binary outcome and its refined version, the one indicating reaching target dose or not, hyperkalaemia and severe hyperkalaemia), treatment effects were first evaluated in the per-protocol (PP) analysis using chi-squred χ^2 or Fisher's exact tests. Permutation versions of these tests was applied for the ITT analyses. These tests work by computing the observed difference in proportions between groups, then repeatedly randomly shuffle treatment labels (who is treated vs control) and recalculate the difference each time. This process recreates a distribution of differences under the null hypothesis, and the p-value is generated by noting where the real observed difference falls in that distribution. The advantages of using permutation tests for ITT include: no need for large sample approximations (unlike χ^2), respects randomization and works well when sample sizes are small and groups are imbalanced.

For better clarity, the paragraph under Statistical Analytical Methods reads now as below:

"For binary and categorical univariate outcomes (the primary binary outcome and its refined version, the one indicating reaching target dose or not, hyperkalaemia and severe *hyperkalaemia), treatment effects were first evaluated using per-protocol (PP) analysis using chi-squared* $\boldsymbol{\chi^2}$ *or Fisher's exact tests.*

Permutation versions of these tests were applied for the ITT analyses. The advantages of using permutation tests for ITT include: no need for large sample approximations, respects randomization and works well when sample sizes are small and groups are imbalanced."

• Further, the statistical analytical method, they state they state that they employed mixed effects models for all biochemical parameters but they do not explain what variables were included in these models.

We apologise that the variables included in the model was not clear – we had relied on the Tables 4 and 6 and thought that they were suggestive. We have now clarified that in the Statistical Analytical Methods section:

"To account for the multiple observations for a participant, associated with measurements on the biochemical markers during the duration of the study (potassium, eGFR, sodium, troponin, NT-proBNP, and ACR), mixed-effects longitudinal models, including random intercepts and random slopes for time with an unstructured variance–covariance matrix, were fitted.. These models were applied to both absolute values and changes over time for these markers, as defined in the Outcomes section. Parameters were estimated using maximum likelihood under MAR assumption for missing observations.

The randomisation process produced two intervention arms with no statistically significant differences in terms of baseline characteristics. Hence, each longitudinal outcome model associated with a biochemical marker included the intervention group and its interaction with time as potential covariates. This has been done with two purposes: to investigate inter-group differences and groups' patterns with time which can be different across groups. If interaction term was not statistically significant, it was excluded from the final models. However, analyses of longitudinal changes (namely the values representing the difference between the current and previous values) were additionally adjusted for corresponding baseline (first visit) marker values to improve estimation efficiency [4]*. ACR was measured twice only, at randomization and at the patient's last visit."*

Similar additions were made for the longitudinal binary models. The section reads now as:

"Longitudinal binary outcomes were analysed using both subject-specific effects using mixed-effects logistic regression models with random intercepts and slopes for time and unstructured structure for the covariance (variance–covariance) using maximum likelihood estimation and population-averaged effects using generalised estimating equations (GEEs). Subject-specific estimates reflect within-individual changes in outcome odds, whereas population-averaged estimates represent mean changes across the trial [5]*. These approaches may differ quantitatively, and they differ in their assumptions about participants attrition—missing at random (MAR) for mixed models and missing completely at random (MCAR) for GEEs—which are not empirically verifiable* [6]. Using a similar argument as for continuous longitudinal outcomes*, given no statistically significant differences, models' covariates included the intervention groups and their interaction with time – that has not been observed to be statistically significant, hence we did not keep the terms in the model. Associations between the outcomes and explanatory variables were assessed by odds ratios (ORs) and their 95% CIs."*

• There is some imbalance in the number of participants randomized to the blinded intervention arms, 53 vs 59. This is not a small difference in a study of just over 100 participants. What is the reason for this imbalance?

We thank the reviewer for this observation. The difference between 53 and 59 participants reflects chance imbalance arising from the 1:1 randomisation process, rather than any departure from the protocol. Randomisation list was produced in Stata and allocation was implemented by the research pharmacist. Because the trial closed before the originally planned sample size of 130 participants was reached, a small numerical imbalance between groups is perfectly plausible. Importantly, baseline characteristics remained broadly comparable between the two groups, consistent with the integrity of the randomisation process. We have added in the *Methods – Randomization* subsection the following paragraph:

"Randomisation list was produced in Stata and allocation was implemented by the research pharmacist. Because the trial closed before the originally planned sample size of 130 participants was reached, a small numerical imbalance between groups is expected. Importantly, baseline characteristics remained broadly comparable between the two groups, consistent with the integrity of the randomisation process."

• I do not understand why the investigators defined study dropout as "absence at a scheduled follow up visit". Does this mean any visit? Would the participant not be allowed to be considered to be still in this study if they drop out by this definition?

Thank you for this question, we agree that the use of the "dropout" terminology can be confusing as it can suggest a patient withdrawal from the study which is not the case. Patients may miss a scheduled visit and may be back for the following one. A missing observation is defined as an observation which should be in the data but for some reason is not. The reference 13 in our manuscript (Sterne et. al.) describes the terminology associated with missing data. We have changed the sentence this reviewer is referring to, and we hope the new semantic brings clarity (Statistical Analytical Methods):

"Patterns of missing observations at the follow-up were examined across treatment groups using a multilevel logistic regression model for a longitudinal binary outcome, where 1 indicates a missing observation and 0 indicates an observed value."

• There is a significant bias introduced in the analysis as 53 SZC and only 43 placebo participants were analyzed for the primary outcome due to lower number randomized and higher number of discontinuations in the placebo group (See CONSORT figure 1).

We thank the reviewer for requesting this clarification. However, we disagree that a significant bias was introduced by this post-randomisation, trial duration events which could have not been envisaged. Although 53 participants in the SZC group and 43 in the placebo group were included in the primary outcome analysis, this difference reflects both the initial randomisation and a higher rate of discontinuation in the placebo group. Importantly, the analysis accounted for missing data using appropriate statistical methods, and the impact of

differential discontinuation was carefully evaluated. These steps help mitigate potential bias and support the robustness of the findings.

• In terms of secondary outcome of achieving target RAASi, the difference barely made statistical significance at the 5% level by ITT.

Thank you for noting that the results of the ITT analysis were consistent with those of the per-protocol analysis, although the strength of the evidence was slightly attenuated.

• The paragraph about discrepancies in inference on page 10 is not understandable. It is rather circuitous and confusing. And ends with a phrase I have never seen in a scientific manuscript: “We have found that, early failures hyperkalaemia (≥5.6mmol/L) for the two patients in the SCZ group would drift away the statistical significance at 0.05 level between the two intervention groups.” What does “drift away from statistical significance mean? It has no meaning to me, please explain.

Thank you for this comment, and we apologise for the lack of clarity in the original wording. The statement referred to a counterfactual (worst-case) sensitivity analysis conducted to assess the robustness of the survival analysis results for hyperkalaemia (≥5.6 mmol/L) in the presence of missing data (Table 5). Our intention was to explore how sensitive the findings were to plausible alternative scenarios regarding missing outcomes. In this context, the original phrasing “drift away from statistical significance” was imprecise and has now been removed. We have revised the text to clarify the interpretation as follows:

“We found that, in a counterfactual scenario in which hyperkalaemia (≥5.6 mmol/L) occurred early during follow-up in the two patients in the SCZ group with missing data, the difference between the intervention groups was no longer statistically significant (Table 5).”

We hope this revised wording improves clarity and better conveys the purpose and findings of the sensitivity analysis.

• The investigators state that a small number of admissions occurred. I assume they mean hospitalizations but it is not clear. And if these are hospitalizations it would help to know whether or not these were hospitalizations for heart failure and if the 2 participants in the SZC who were admitted were admitted for heart failure. And if they were heart failure hospitalizations it would be of interest to know dose of SZC they were on at the time of admission.

Thank you very much for your question regarding admissions in the study population. There were a total of 13 hospital admissions, of which 2 occurred in the SZC group. Table S3 provides details of the hospital admissions and the reasons for each admission.

We have added the following sentence to the second-to-last paragraph of the Results section:

“*There was one hospital admission due to heart failure in the intervention group and three in the placebo group. The reasons for admissions are shown in Table S5.*”

Both participants in the intervention group were receiving 5 g of SZC at the time of admission.

Table S6: The cause of admission during follow up

Admission Causes	Intervention	Placebo	Grand
Arrhythmia		3	3
Blood Pressure		1	1
Elective Insertion of ICD		1	1
Electrolytes		2	2
Falls		1	1
Heart Failure,	1	3	4
ICD problems		1	1
UTI, with renal calculus		1	1
NSTEMI	1		1
Grand Total	2	13	15

Legend: Admission causes for severe adverse events within trial stratified by treatment arm. *ICD – implantable cardiac defibrillator; NSTEMI – non-ST elevation myocardial infarction; UTI – urinary tract infection*

• In the discussion the investigators claim the strength of their study is that it is “real-world” population. I challenge this. What is different from other randomized double-blind placebo controlled trials in HF and CKD populations. This was not a pragmatic trial. Each participant was approached individually and strict inclusion and exclusion criteria applied. I think it is misleading to suggest this is a “real-world” trial. Many studies in people with HF and CKD include older patient populations.

We thank the reviewer for this comment and agree that this randomized controlled trial included patients based on strict inclusion criteria, and therefore may differ from populations typically seen in routine clinical practice.

We would, however, like to emphasize that, in contrast to other studies (e.g., Realise-K), our study included patients with more advanced kidney disease. In Realise-K, the mean estimated glomerular filtration rate (eGFR) at screening was 58±19 ml/min/1.73 m², whereas in the LIFT study the mean baseline eGFR was 35±12 ml/min/1.73 m².

We have revised the fifth paragraph of the Discussion section as follows: “*A key strength of this study is its unique trial population of elderly patients with advanced kidney disease. The mean eGFR was 35±12 ml/min/1.73 m², and the median age was 73 (range 38–92) years in the LIFT study, compared with 58±19 ml/min/1.73 m² and 70 (range 36–95) years in Realise-K.”*

• The manuscript does not support the specific statement made in the conclusion. Specifically, the investigators do not quantify “rapid uptitration” as the paper provides no data on the rate of uptitration of RAASi. There is no data nor mention of rate of uptitration in the entire manuscript.

We thank the reviewer and agree on the importance of clarifying the protocol for up titration of RAASi. The up-titration was done by study doctors following a protocol shown in Table S1 and placebo/SZC dose was changed following Table S2, provided it was safe and well tolerated. The speed of changes can be appreciated from the figures below showing the

different doses at different visits of the study as below. The protocol was similar to the "Strong HF" study.

Please see Figure S1 A and B : The supplementary figures show the up titration of ARNI/ACEi/ARB and MRA in visits 1-7 two weeks apart with clinical and biochemical evaluation how the dose changed in with SZC and Placebo.

Minor

• The investigators should comment on the fact that only 25% of the study population were female. A 3:1 ratio of male:female for HFrEF is not what is generally observed in the broader population.

Thank you for this highlighting this important question.

When designing a study, a key principle is that the analytical sample should adequately represent the population to which inferences are intended to apply. In this case, the target population comprises hospitalized patients with chronic kidney disease and heart failure with reduced ejection fraction. The composition and structure of this population remain poorly characterized. However, a recent observational study conducted in our large, specialized CKD–HF clinic—including consecutive patients with CKD stages 3–5 and heart failure seen between 12 September 2019 and 11 November 2021, comprising 667 admissions from 318 patients—demonstrated a similar sex distribution. Specifically, our 2025 publication in Cardiorenal Medicine (https://doi.org/10.1159/000541806) reported 109 (34.3%) women and 209 (65.7%) men in the background cohort (Table 1).[7] In light of these findings, we consider our analytical sample to be broadly representative of this population, particularly when further restricted to patients with reduced ejection fraction. We have added a sentence at the end of the discussion where we cited the paper above.

"The sex distribution observed in our sample is consistent with that reported in a recent CKD–HF clinic cohort and remains plausible when further restricted to patients with reduced ejection fraction, supporting the representativeness of our study population."

• It would be helpful to list the distribution of participants by stage of CKD in Table 1.

We have added the requested information (displayed below) in the Table 1.

CKD Stage	Intervention	Placebo
CKD 3a	10 (18.9%)	15 (25%)
CKD 3b	26 (49.1%)	22 (37%)
CKD 4	15 (28.3%)	22 (37%)
CKD 5	2 (3.8%)	0 (0%)

• There are no figure legends for the plethora of plots starting on page 28 of the manuscript.

Thank you - we apologise that we have omitted figures' legends, probably because they have been uploaded as .eps files which to not accept legends. We have now added the Figures' legends at the end of the tables section, without the figures which are uploaded as requested by the journal.

Reviewer #2 (Remarks to the Author):

This is an interesting study. I have the following comments.

Methods

p.5, line 104. It stated that "… by the study pharmacist according to the randomisation schedule". Please provide more details about the randomisation schedule.

Thank you, we have changed the sentence in the *Randomisation* section for better clarity:

"Eligible patients were enrolled by study clinicians and subsequently assigned to treatment groups by the study pharmacist according to a 1:1 randomisation schedule generated using a computer-based random number sequence. The allocation sequence was not accessible to the enrolling clinicians or patients, and treatment assignment was implemented by the pharmacist to ensure allocation concealment."

For the mixed effects logistic regression models, please indicate clearly about the random effects structure, the fixed effects, and the estimation methods used in the models. What're the covariates included in the GEEs models?

Thank you for requesting this clarification. We have used a mixed-effects logistic regression model with random intercepts and slopes for time and unstructured setting for the covariance (variance–covariance). However, the results when considering the simplest covariance structure, namely independent, were not qualitatively different and quantitatively very close in terms of standard errors. The estimation procedure was that of maximum likelihood. The assumption on the missing observations was that of missing at random (MAR).

Given the statistically equivalent groups due to randomisation, the main interest was to use the models to understand the potential treatment effect and their interaction with time on these longitudinal outcomes. No interaction between groups and time was statistically significant and it has been consequently dropped from the model.

Models on longitudinal changes have been additionally adjusted for the baseline (first visit) values as it is usually the case in the analysis of change. Even no evidence for differences in the baseline values is observed, in the context of randomised clinical trials, – estimates' precision may be gained.[4]

The Statistical Analytical Methods section were altered to improve clarity as requested by the reviewer:

"Longitudinal binary outcomes were analysed using both subject-specific effects using mixed-effects logistic regression models model with random intercepts and slopes for time and unstructured setting for the covariance (variance–covariance) using maximum likelihood estimation and population-averaged effects using generalised estimating equations (GEEs). Subject-specific estimates reflect within-individual changes in outcome odds, whereas population-averaged estimates represent mean changes at group level across the trial. These approaches may differ quantitatively, and they differ in their assumptions about participants attrition—missing at random (MAR) for mixed models and missing completely at random (MCAR) for GEEs—which are not empirically verifiable"

Using a similar argument as for continuous longitudinal outcomes, given the intervention groups statistical equivalence, models' covariates included the intervention arms indicator and their interaction with time.

"Given the intervention groups statistical equivalence, models' covariates included the intervention groups and their interaction with time – that has not been observed to be statistically significant hence we did not keep the terms in the model. Associations between the outcomes and explanatory variables were assessed by odds ratios (ORs) and their 95% CIs."

It's not clear the covariates used in the Cox-regression models.

Thank you, we have changed the sentence in the *Statistical Analytical Section* for better clarity:

"Cox proportional hazards models were also fitted on intervention groups variable only, with proportionality assumptions formally assessed. Associations between outcomes and group variable were measured by hazard ratios (HRs) and their corresponding 95%CIs."

P.7, lines 155-158. It looks that both Poisson and negative binomial regression models were used for analysing count data in the analysis. Suggest using the one that provides more reliable results.

Thank you. We aimed to pre-empt a potential request to use negative binomial regression. We initially fitted negative binomial models to assess the presence of overdispersion; however, no evidence of overdispersion was observed. Therefore, the final analyses were conducted using Poisson regression models with robust variance estimation. Given the similar interpretation of the two approaches in this context, inference was based on the Poisson models. The following sentence has been added to the manuscript to enhance clarity:

"*Negative binomial regression was used to assess potential overdispersion - which hasn't been observed."*

P.7, line 169. "Mixed effects models were also conducted for all biochemical markers such Potassium, eGFR,…". Suggest specifying the models, such as linear mixed effects models if that's the case.

Thank you – the statement has been improved for clarity. Unlike longitudinal binary outcomes, in case of longitudinal continuous outcomes, maximum likelihood estimation and GEE estimation results in very similar estimates. Reference number 14 in the paper offer more details for research but detailing here would be beyond the scope of this paper.

We have changed the section in the *Statistical Analytical Section* for better clarity:

"Mixed-effects longitudinal models, including random intercepts and random slopes for time with an unstructured variance–covariance matrix, were fitted for all biochemical markers (potassium, eGFR, sodium, troponin, NT-proBNP, and ACR). These models were applied to both absolute values and changes over time, as defined in the Outcomes section.

Parameters were estimated using maximum likelihood under MAR assumption for missing observations. All models included the intervention group and its interaction with time; however, as the interaction term was not statistically significant, it was excluded from the final models. Analyses of longitudinal changes were additionally adjusted for baseline (first visit) values of the corresponding outcome to improve estimation efficiency."

Results

P.10, line 241-242. "0.02 mmol/L (95% CI 0.00–0.05)". Suggest changing 0.00 to 0.002 as shown in the table.

Thank you – we have done this.

P.10, line 245. "95% CI 0.0001–0.06". 0.0001 seems to be 0.001. Please check.

That is correct – thank you

Table 1 Please add the number of participants in sodium zirconium group and keep variables (mean and SD) in the same format within the table.

Thank you – that should be certainly the case. We have replaced ± with forward slash when it was the case.

Table 2 Note that the primary outcomes were analysed using both per-protocol and intention-to-treat (ITT) approaches. This's a superiority study to compare differences between two randomised groups. The ITT principle is preferred approach for analysing the data. Caution should be made when interpreting the results based on "per protocol" analysis. In addition, the meaning of "Reached Potassium 5.6" was not clear.

Thank you. To our best knowledge, clinical trials always require the PP analyses first in tandem with their ITT version. We have added in the Table 2 legend. We think we exhibited caution when interpreting the PP results – nevertheless, the ITT analyses showed consistent results. We have also conducted considerable sensitivity analyses to the missing data too to ensure robustness of the findings.

"*Reached potassium 5.6 (or 6) means that patient presented 5.6 (or 6) mmol/L at least once during the follow-up.*"

Table 3. All abbreviations need to be clearly defined in the table.

Thank you, we have added a sentence in the legend for clarity, and we have replaced OBS with OBSERVATIONS.

"OR stands for ODDS ratios estimated for each scenario – presented with their p-values and 95% confidence intervals (CI), SZC – sodium zirconium."

Table 4. Please indicate the covariates used the models.

Thank you – we have added extra clarification in the Table 4 legend.

“Covariates include time and intervention group variable-no interaction between the two has been observed in any of the models. In addition, the models fitted on the longitudinal Potassium changes were also adjusted for their baseline (first visit) values.”

Table 5. In the table, different versions of the log-rank test were presented. It would be better to select the most appropriate ones.

Thank you – it is true. However, given the missing data context we felt that showing various consistent tests and analyses would add to the robustness of the results for 5.6 mmol/L hyperkalaemia. As for 6 mmol/L hyperkalaemia, the events are much less in numbers (Table 2) and the exploratory analyses using Cox regression which is based on conditional likelihood estimation show some inconsistencies in terms of statistical significance.

Reviewer #3 (Remarks to the Author):

To revise a couple of trials and cite them

Banerjee et al. report data from their double blind placebo controlled to test whether treatment with a potassium binder (SZC) resulted in lower rates of hyperkalemia and better uptitration of HF medication in patients with impaired renal function. Overall this is an interesting topic, although not completely novel. Several trials have been executed to test this hypothesis (i.e. uptitration RAASi in patients with HF): including PEARL, DIAMOND and REALIZE-K, only the latter one is mentioned in the manuscript. Furthermore, there are concerns that the sodium load in ZSC may deteriorate HF (as has been observed in the the REALIZE-K trial.
In the current trial the authors find that SZC leads to more uptitration and less hyperK.

The analyses, tables and figure are overwhelming and not very clear. Also the combination of reporting of ITT and per protocol in the same paragraphs are confusing. The paper would benefit from a clearer presentation, including less tables and a focus on the main outcome. The drop-out is relatively high. I miss a paragraph on safety.

We thank this reviewer for their thoughtful and constructive comments, as well as for their clear appreciation of the clinical importance and applicability of our research. The additional tables are in supplement. We have moved 3 tables from the manuscript to the supplement. We agree that there are previous trials of RAASi maximisation, such and PEARL and DIAMOND of patients with higher GFR than the present study.

We have now included the other RAASi maximisation studies in discussion. The changes in the manuscript are as follows:

"*An initial trial in 105 heart failure patients with CKD or history of hyperkalaemia, the maximal dose of spironolactone i.e 50 mg daily could be maintained in 91% patients with serum potassium less than 5.5 mmol/L with RLY5016 versus 74% (p=0.019) with placebo, but the eGFR at baseline in the intervention arm was higher than the present study 84±35 ml/min/1.73m2.* [8]

A larger trial of heart failure and CKD the same agent patiromer, while maintaining half maximal dose of ACEi/ARB/ARNI maximal dose of spironolactone or eplerenone i.e 50 mg daily, was maintained in 86% versus 81% with placebo (HR 0.62; 95% CI 0.45, 0.87; P = 0.006) in maintaining normokalaemia however the eGFR at baseline was 62.6±22.6 ml/min/1.73m2.[9]

Specific comments:

The authors mention that a power calculation was published previously, however it is crucial to include a summary in the main paper.

Very many thanks for this request. We have now included a subsection *Sample Size Calculation* under *Methods* whereby we inform the reader with the published protocol content:

"Group sample sizes of 52 will achieve approximately 90% power to detect a difference between the group proportions of 0.3 using a two-sided Z-test with pooled variance and a two-sided χ2 test. The proportion of responders in the placebo arm is assumed to be 0.5 with the corresponding proportion in the active arm 0.8 under the alternative hypothesis. The statistical significance reference level of a test is 0.05. This results in a total of 104 patients required and, accounting for 20% loss to follow-up, we will recruit 130 patients to be randomised to each intervention group. Further details can be found in the published protocol".

How did the authors deal with the two components of primary endpoint: target dose RAASi and K<5.6? Was this prespecified? Do the authors have a statistical analysis plan?

Thank you for requesting clarity on the primary end-point and statistical plan. We have now revised and restructured the Methods section into clearer subsections, including *Study design*, *Patients*, *Intervention*, *Randomization*, *Outcomes' definitions*, *Sample size calculation* (added in response to the reviewer's suggestion), and *Statistical Analytical Methods*.

The *Outcomes' Definitions* subsection contains these definitions which we hope enhanced clarity:

"The primary endpoint was defined as a composite binary outcome, with success defined as achieving both the target RAASi dose and maintaining a serum potassium concentration <5.6 mmol/L throughout the trial. Failure was defined as either not achieving the target RAASi dose or achieving it in the presence of a serum potassium concentration reaching 5.6 mmol/L or higher.

A refined four-level categorical response was defined by jointly classifying the target dose achievement (YES or NO) and hyperkalaemia status (serum potassium ≥5.6 mmol/L or <5.6 mmol/L).

Three other different secondary binary outcomes were defined for (i) achievement of the target RAASi dose or not, (ii) occurrence of hyperkalaemia (serum potassium ≥5.6 mmol/L) or not, and (iii) occurrence of severe hyperkalaemia (serum potassium ≥6.0 mmol/L) or not."

More information on the statistical plan now appear in the "Statistical analytical methods" in addition to the published protocol.

How was the RAASi uptitration protocol defined?

We thank the reviewer for requesting us to clarify the RAASi up-titration protocol which is extremely important for this elderly high population, as a possible guide to implement the results from our study. We have added the following in the methods section, intervention subsection:

"At each study visit, two weeks apart, the treating physician—blinded to treatment allocation—was instructed to up-titrate RAAS inhibitors according to the study protocol, progressing from the starting dose to the half maximal dose and subsequently to the maximum dose at two-week intervals, provided it was safe to do so on clinical and

biochemical evaluation. Up-titration was undertaken provided that patients were not hypotensive (systolic blood pressure <90 mmHg), had no symptomatic or clinically significant postural drop in systolic blood pressure (≥20 mmHg), were not hyperkalaemic, and had not experienced significant adverse effects at the previous dose.

The starting, half maximal, and maximum doses of ARNI, ACE inhibitors, ARBs, and MRAs are detailed in Table S2. At each visit, potassium levels were assessed using both an urgent point-of-care test and a standard laboratory measurement. The dose of placebo or sodium zirconium cyclosilicate was adjusted according to the study protocol. Specifically, treatment was withheld when serum potassium was <3.5 mmol/L and increased when potassium exceeded 5.0 mmol/L, as detailed in Table S1. Table S3 outlines the recommended dose escalation for patients not receiving any RAAS inhibitors at baseline and Table S5 shows the specific RAASi agents used."

Target RAASi dosing was defined according to the LIFT trial protocol, based on medication type and dose, from less than half maximal dose to more than or equal to half maximal dose OR from half dose to maximal dose.

Table S2: The minimum, half-maximal, and maximum dose of ACEi/ARB/ARNI and MRA

	**Minimum dose**	**Half maximal dose**	**Maximum dose**
**ACEi/ARB/ARNI (one of the following)**			
Ramipril	2.5 mg OD	5 mg OD	10 mg OD
Perindopril arginine	2.5 mg OD	5 mg OD	10 mg OD
Enalapril	2.5 mg OD	5 mg OD	10/20/40 mg OD
Lisinopril	2.5 mg OD	5 / 10 mg OD	20 / 40 mg OD
Perindopril erbumine	2 mg OD	4 mg OD	8 / 16 mg OD
Sacubitril/valsartan	24 / 26 mg BD	49 / 51 mg BD	97 / 103 mg BD
Losartan	25 mg OD	50 mg OD	100 mg OD
Candesartan	4 mg OD	8 / 16 mg OD	32 mg OD
Irbesartan	75 mg OD	150 mg OD	300 mg OD
Telmisartan	20 mg OD	40 mg OD	80 mg OD
Olmesartan	10 mg OD	20 mg OD	40 mg OD
Valsartan	40 mg BD	80 mg BD	160 mg BD
**MRA (one of the following)**			
Spironolactone	12.5 mg OD	25 mg OD	50 mg OD
Eplerenone	12.5 mg OD	25 mg OD	50 mg OD

Legend: The study clinician was instructed to increase from starting, to half-maximal, and maximum dose provided it was safe to do so. See table S3 for more information. BD – twice a day; OD – once a day; ARNI – Angiotensin receptor neprilysin inhibitor; MRA – mineralocorticoid receptor antagonist.

Table S1: Instructions for changing the dose of IMP at every 2-weekly study visits

SZC or Placebo dose at start of visit	Serum potassium end of visit	Dose change at the end of visit
If on 10 g daily	> 5.0 mmol/L	Continue 10 g daily

	(but ≤ 6.0 mmol/L)	
	3.5–5.0 mmol/L	Change to 5 g daily
	< 3.5 mmol/L	Stop
	> 6.0 mmol/L	Stop and initiate rescue
If on 5 g daily	> 5.0 mmol/L	Change to 10 g daily
	3.5–5.0 mmol/L	Continue 5 g daily
	< 3.5 mmol/L	Stop
If on 0 g daily (temporary stop)	≤ 5.0 mmol/L	Continue with no IMP
	> 5.0 mmol/L	Initiate 5 g daily

Legend: The IMP (SZC or placebo) was changed depending on serum potassium and previous dose of IMP. IMP – investigational medicinal product; SZC – sodium zirconium cyclosilicate

Table S3: Guidance on dose escalation of RASi (ACEi/ARB/ARNI) and MRA in 2-weekly study visits

	Ideal Doses*						
Visits	Visit 1	Visit 2	Visit 3	Visit 4	Visit 5	Visit 6	Visit 7
RASi (ACEi/ARB/ARNI) (one of the following)							
Sacubitril/Valsartan *(Twice a day)*	0mg	24/26mg	24/26 mg	49/51 mg	49/51 mg	97/103 mg	97/103 mg
Ramipril *(once a day)*	0mg	2.5mg	2.5 mg	5mg	5mg	10 mg	10 mg
Candesartan *(once a day)*	0mg	4 mg	4 mg	8 mg	16 mg	32 mg	32 mg
		Minimum dose		Half maximal dose		Maximum dose	
MRA (one of the following)							
Eplerenone (Once a day)	0mg	0mg	12.5mg	12.5mg	25mg	25mg	50 mg
Spironolactone (Once a day)	0mg	0mg	12.5mg	12.5mg	25 mg	25 mg	50 mg
			Minimum dose		Half maximal dose		Maximum dose

Legend: Table showing how dose could have been escalated in a patient starting with no RAASi where maximum dose can be achieved over 7 study visits. *RASi – renin-angiotensin system inhibitor; MRA – mineralocorticoid receptor antagonist*

It is unclear which dose of RASi / MRA patients were using at the moment of randomization.

This should be in the baseline table. It is also relevant to report this in the outcome section: i.e. dose ACEi achieved in both groups; MRA dose achieved; were ARNI's prescribed?

We thank the reviewer in requesting the dose of ARNI/ARB/ACEi at baseline and follow up. ARNI was used in majority of the patients. We have demonstrated the data in table S54 showing the agents used in baseline and figures showing the doses at different visits (Figure S1 A and B)

Table S5: Specific agents used during the study in all patients

Agent	At baseline	At study end
Sacubitril/Valsartan	83	82
Ramipril	16	13
Candesartan	8	7
Losartan	1	1
Irbesartan		1
Eplerenone	37	58
Spironolactone	19	27

Legend: Table shows the specific RASi and MRA agents used within the trial at the start of study and study end. *RASi – renin-angiotensin system inhibitor; MRA – mineralocorticoid receptor antagonist*

Figure S1: RAASi dose in each visit during the study

Figure S1 A and B: The dose of ACEi/ARB/ARNI (A); The dose of MRA in 2-weekly study visits (B) : The bars indicate the number of participants on none, minimum dose, half maximal, and maximum dose of RASi and MRA in visits 1-7 two weeks apart stratified by SZC and Placebo arms. *RASi – renin-angiotensin system inhibitor; MRA – mineralocorticoid receptor antagonist; SZC – sodium zirconium cyclosilicate*

A

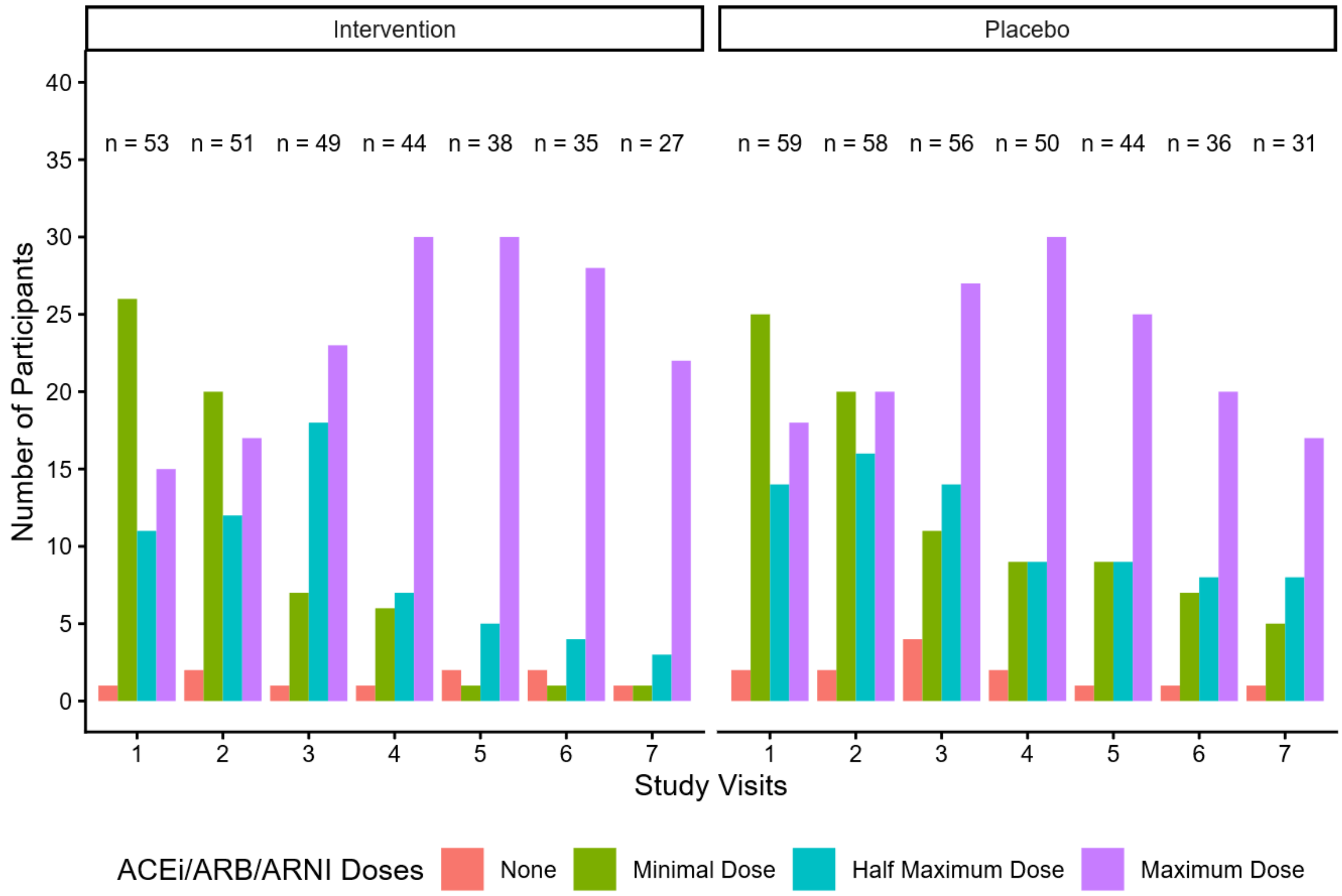

ACEi/ARB/ARNI Escalation

B

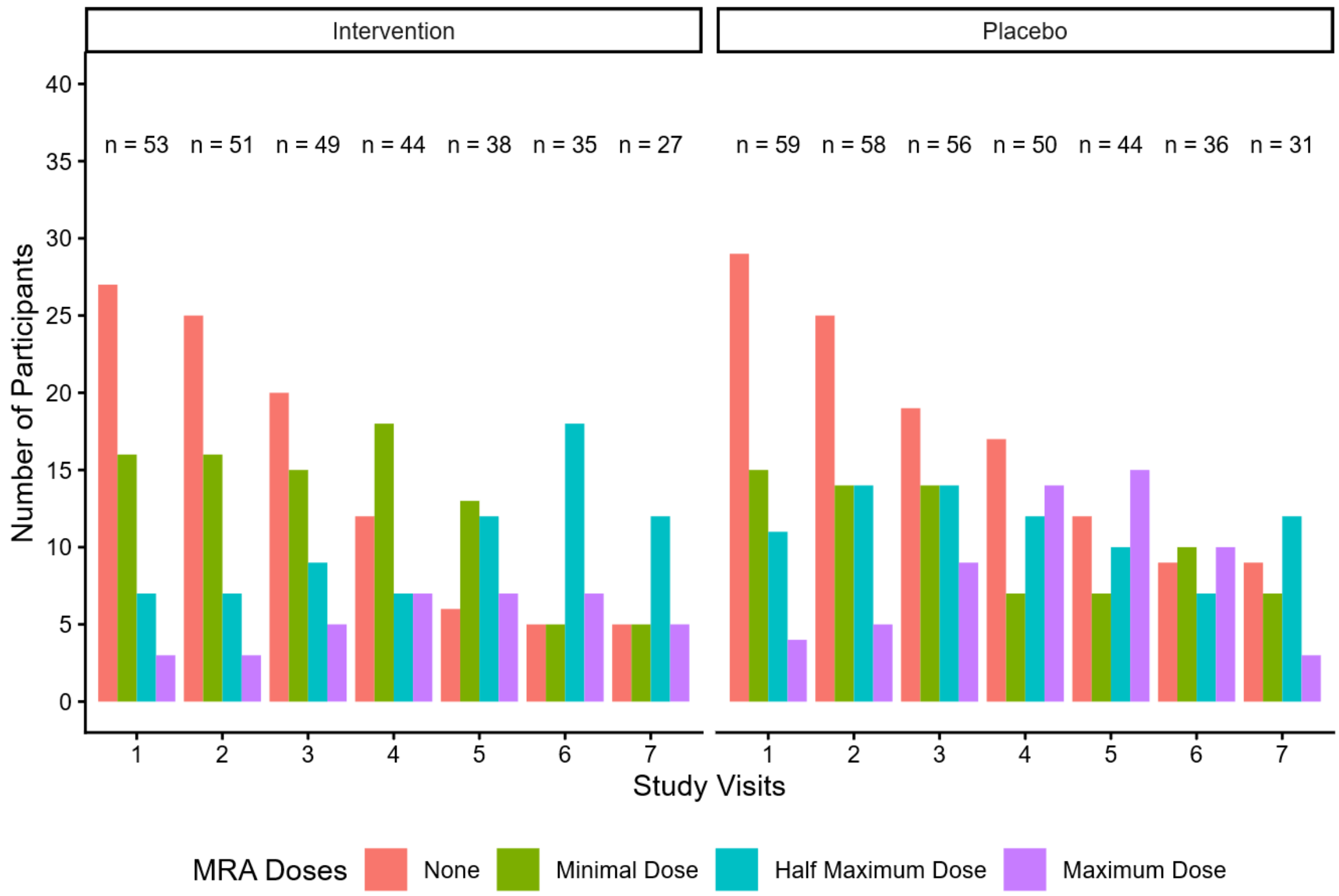

MRA Escalation

From the methods section it is unclear what the author mean with “Final RAASi dosing was determined using a tailored, semi-automated Stata script developed and implemented by the

study statistician in collaboration with the clinical lead". What kind of script are the authors referring to?

We thank the reviewer for highlighting this important point. We feel that we need now to clarify that treatment decisions during the trial were made by clinicians in accordance with the study protocol see above and table S2 &S3, whereas the Stata routine was applied after data collection to consistently map observed treatment histories onto the predefined outcome categories for analysis. This distinction reflects the role of the script as an analytical implementation of the protocol rather than a clinical decision-making tool.

We have revised the Methods section to provide a clearer and more detailed description of how the final RAASi dose was derived from the protocol-defined algorithm (Figure 1, Table S2, and Table S3), including the classification of medication-specific target doses.

How was target dose defined? Is there evidence that 25mg spironolactone is superior to 50mg?

The European Society of Cardiology Clinical Practice Guidelines (and British National Formulary) recommends a maximum dose of both spironolactone and eplerenone as 50 mg daily for heart failure and that is how the maximal dose of MRA defined in the study. Previous RAASi maximisation studies (PEARL, DIAMOND, REALIZE-K) also defined 50 mg daily of MRA as the maximum dose. There is no well powered study of spironolactone in heart failure comparing the two doses 25 mg and 50 mg daily.

The difference between previous trials with potassium binders to enable uptitration of HF medication is that patients with eGFR<30 were allowed to participate. As such it would be important to better specify this subgroup.

Thank you for highlighting the uniqueness and the challenge of our population characteristics. We have added a sentence at the end of the *Methods – Patients* subsection which reads as below:

"Our population is unique in that it includes patients with advanced chronic kidney disease with eGFR<30 and very prone to AKI and hyperkalaemia."

The numbers of patients in the advanced stages of CKD are in table one. We did not do a subgroup analysis as it was not planned in our protocol.

Results section:

Please include linear troponin and NTproBNP data (line 178); log scale makes them uninterpretable to a clinician.

We thank the reviewer. We have added this in the results section first paragraph "*Overall baseline laboratory markers included potassium 4.98±0.38 mmol/L, eGFR 35±12ml/min/1.73m^2, Troponin 31 (IQR 21 – 52) ng/L, NT-pro BNP 1683 (IQR 720, 4102)*

pg/L…"

It is confusing to report in the same paragraph the ITT and per-protocol analysis. As a sensitivity analysis per-protocol can be mentioned at the end of the results section.

Thank you for the comment. We wish to highlight both PP and ITT results' consistently as we believe that this is useful for our readers. The PP analysis is always required, as PP plays the role of *complete data* analysis whilst the ITT analysis corresponds to *observed data* analysis (Multiple imputation for missing data in epidemiological and clinical research: potential and pitfalls | The BMJ). [6]

Drop out was double for the SZC group (10% vs 19%). What was the reason for drop out?

We recognise the dropout percentages in each intervention group. However, the difference in proportions is not statistically significant as mentioned at the end of the first paragraph of the results.

"Six out of 59 patients (10%) in the placebo group and 10 out of 53 (19%) patients in the SZC group withdrew from study and there was no significant difference between the distribution of the withdrawals between the two groups (p=0.189)."

Moreover, at the end of the Results section we mentioned the reasons for patients' withdrawal which were either based on clinical decision and the same number based on their own decision.

" Supplementary Tables S7 and S8 investigated patterns in patients' withdrawal and missing observations. In the placebo group, 3(5%) of the participants withdrew based on clinical decision and the same number based on their own decision. In the intervention group, 2(4%) of the participants withdrew based on clinical decision and 8(15%) based on their own decision. There was no evidence to support any difference between the distribution of the attrition numbers across the two groups (P=0.209)."

The authors report (line 187) 24 of 43 patients, but in the itt population this should be 53. What happened to the other 10 patients? Again, the primary outcome should be ITT.

Thank you for noting that 10 patients were withdrawn. We are sorry that our statements were not fully clear to the reviewer and we are not entirely sure what "the primary outcome should be ITT" really means.

Table 2 present both per protocol (PP) and intention to treat (ITT) analyses for cross sectionally defined outcome including the primary outcome, as customary in clinical trials with missing data and patient withdrawal. The sentence the reviewer refers to represents the result of the PP analysis, and it includes the p values for both ITT and PP analysis. The adjacent column (the last) presents the corresponding ITT analysis which include all 112 participants (specified in the column head) and is based on randomization or permutation tests corresponding to binary outcomes. These tests, applicable because of a priory randomization process, test the null hypothesis of exchangeability under no treatment effect. In other words, if there is no treatment effect, allocation of labels (0/1) is equally plausible across groups. The ITT analysis of the primary outcome is consistent with the PP analysis result.

Why not report % diabetes, %hypertension. Please report only IQR is non-normally distributed. Providing both mean and IQR makes it overly complicated to read.

Thank you very much for your comments. Table 1 presents the percentages of diabetes, hypertension, IHD history, smoking, obesity and hypercholesterolemia stratified by intervention groups as it is customary in clinical trials.

From our experience, presenting highly skewed data solely as median and IQR can make it difficult for researchers to use these results in sample size calculations, in future. Therefore, we have reported both Mean (SD) and Median (IQR) In addition, we have also provided log-transformed results to support scientific and methodological interpretation.

Figure 1; difficult to read: what does potassium 5.6 Yes mean (below 5.6? above?)

Thank you for highlighting this issue – we have now clarified in the legend of relabelled Figure 2 the meaning of potassium 5.6 Yes/No and Table 2 legend the meaning of Reached Potassium 5.6 or 6

“Reached potassium 5.6 (or 6) means that patient presented 5.6 (or 6) mmol/L at least once during the follow-up.”

We have now also added Figures’ legends - for which we apologise for forgetting to add in the first instance.

References

1. Mebazaa, A. *et al*. Safety, tolerability and efficacy of up-titration of guideline-directed medical therapies for acute heart failure (STRONG-HF): a multinational, open-label, randomised, trial. *Lancet* **400**, 1938–1952 (2022).

2. Murphy, D., Ster, I. C., Kaski, J. C., Anderson, L. & Banerjee, D. The LIFT trial: study protocol for a double-blind, randomised, placebo-controlled trial of K(+)-binder Lokelma for maximisation of RAAS inhibition in CKD patients with heart failure. *BMC Nephrol.* **22**, 254–2 (2021).

3. Heidenreich, P. A. *et al*. 2022 AHA/ACC/HFSA Guideline for the Management of Heart Failure: A Report of the American College of Cardiology/American Heart Association Joint Committee on Clinical Practice Guidelines. *J. Am. Coll. Cardiol.* **79**, e263–e421 (2022).

4. Senn, S. Change from baseline and analysis of covariance revisited. *Stat. Med.* **25**, 4334–4344 (2006).

5. Zeger, S. L. & Liang, K. An overview of methods for the analysis of longitudinal data. *Statistics in Medicine* **11**, 1825–1839 (1992).

6. Sterne, J. A. C. *et al*. Multiple imputation for missing data in epidemiological and clinical research: potential and pitfalls. *BMJ* **338**, b2393 (2009).

7. Parmar, S. *et al*. Risk of Hospital Admissions and Death in Patients with Heart Failure and Chronic Kidney Disease: Findings from a Novel Multidisciplinary Clinic. *Cardiorenal Med.* **15**, 249–260 (2025).

8. Pitt, B. *et al*. Evaluation of the efficacy and safety of RLY5016, a polymeric potassium binder, in a double-blind, placebo-controlled study in patients with chronic heart failure (the PEARL-HF) trial. *Eur. Heart J.* **32**, 820–828 (2011).

9. Butler, J. *et al*. Patiromer for the management of hyperkalemia in heart failure with reduced ejection fraction: the DIAMOND trial. *Eur. Heart J.* **43**, 4362–4373 (2022).

Response to reviewers NCOMMS-25-97999B

Title: Rapid Renin Angiotensin Aldosterone System inhibitor optimisation in patients with chronic kidney disease and heart failure with reduced ejection fraction using potassium binder; a randomised, double-blinded, placebo-controlled trial

We thank all reviewers for their help which has hugely improved the quality of the manuscript. We have responded to their comments point by point below.

Reviewer 1:

Comment: The manuscript is substantially improved and the authors responses are satisfactory.

Response: Thank you for mentioning that we have responded to the queries as well.

Reviewer 2:

Comment: The authors have responded well to my questions. Noted that the same methods for analysing biochemical markers (potassium, eGFR, sodium, troponin, NT-proBNP, and ACR) were described repeatedly in the statistical methods on page 8 and 11

Response: Many thanks for highlighting the reputation of the method for analysis of bio markers in pages 8 and 11. We have now removed the sentence mixed effect language models… ACR from page 11 paragraph 2.

Reviewer 3:

Comment: The authors responded to all my comments. I have no additional comments, but I think the manuscript would benefit from removing the per-protocol analysis/results to the supplements

Response: We thank the reviewer for the thorough review. We have retained the per protocol analysis for the benefit of the readers; for the reasons that we have mentioned in our initial response to the reviewer.
