# Supplementary material for: Rapid Renin Angiotensin Aldosterone System inhibitor optimisation in patients with chronic kidney disease and heart failure with reduced ejection fraction using potassium binder: a randomised, double-blinded, placebo-controlled trial
Source: Nat Commun. 2026 Oct 14;17:11614. doi: 10.1038/s41467-026-78377-0 (PMC13754891; doi:10.1038/s41467-026-78377-0)
Supplement: Supplementary file 3 [file 41467_2026_78377_MOESM1_ESM.docx]

## Nature Communications manuscript NCOMMS-25-97999-T

Title: “Rapid Renin Angiotensin Aldosterone System inhibitor optimisation in patients with chronic kidney disease and heart failure with reduced ejection fraction using potassium binder; a randomised, double-blinded, placebo-controlled trial”

## Supplementary documents

## Supplementary table S1

Table S1: Baseline characteristics of trial participants at the point of randomization, including laboratory measurements
Legend: Categorical and binary variables are presented as numbers and percentages, while continuous variables are summarized using means, standard deviations, medians and interquartile ranges. Summaries are presented stratified by the two-intervention group. Abbreviations: NTproBNP = N terminal-pro-brain-natriuretic peptide; ACEi/ARB/ARNI= angiotensin-converting-enzyme inhibitor/ angiotensin-receptor blocker/ angiotensin-receptor-neprilysin inhibitor; MRA= mineralocorticoid receptor antagonist; SGLT2i=sodium-glucose-transporter 2 inhibitor.

| Baseline Characteristics | Summaries/Categories | Placebo (n=59) | Sodium Zirconium (n=53) |
| --- | --- | --- | --- |
| DEMOGRAPHICS | | | |
| Age (years) | Mean/SD | 72.59/10.64 | 73.87/11.62 |
|  | Median (Q1-Q3) | 73(66-80) | 76(69-81) |
| Gender | Female | 15 (25%) | 15 (28%) |
|  | Male | 44(75%) | 38(72%) |
| Ethnicity | White | 41 (69%) | 34 (64%) |
|  | non-White | 18(31%) | 19(36%) |
| ASSOCIATED CHRONIC CONDITIONS | | | |
| Diabetes | Yes | 29 (49%) | 32 (60%) |
|  | No | 30(51%) | 30(40%) |
| Hypertension | Yes | 35 (59%) | 25 (47%) |
|  | No | 24(41%) | 28(53%) |
| IHD history | Yes | 34 (58%) | 35 (66%) |
|  | No | 25(42%) | 17(32%) |
|  | Missing | 0(0%) | 1 (2%) |
| Smoker | Ever | 34 (58%) | 36 (68%) |
|  | Never | 25 (42%) | 17 (32%) |
| Obesity | Yes | 15 (25%) | 16 (30%) |
|  | No | 44 (75%) | 37(70%) |
| Hypercholesterolemia | Yes | 32 (54%) | 27 (51%) |
|  | No | 27(46%) | 24(45%) |
|  | Missing | 0(0%) | 2 (4%) |
| CKD Stage | CKD 3a | 15 (25%) | 10 (18.9%) |
|  | CKD 3b | 22 (37%) | 26 (49.1%) |
|  | CKD 4 | 22 (37%) | 15 (28.3%) |
|  | CKD 5 | 0 (0%) | 2 (3.8%) |
| LABORATORY MARKERS | | | |
| Potassium levels (baseline) | Mean/SD | 4.93/0.36 | 5.03/0.39 |
|  | Median (Q1-Q3) | 5(4.7- 5.1) | 5(4.8- 5.2) |
| eGFR (baseline) | Mean/SD | 36.17/11.59 | 34.72/11.96 |
|  | Median (Q1-Q3) | 34(27- 45) | 33(28- 41) |
| Sodium | Mean/SD | 138.97/2.99 | 138.75/3.29 |
|  | Median (Q1-Q3) | 139(138- 141) | 139(137- 140) |
| Troponin (log scale) | Mean/SD | 3.49/0.71 | 3.50/0.74 |
|  | Median (Q1-Q3) | 3.38(3.09- 3.81) | 3.56(2.97- 4.02) |
| Troponin (original scale) | Mean/SD | 47.03/75.94 | 43.81/39.79 |
|  | Median (Q1-Q3) | 29.5(22, 45) | 35(19.5, 55.5) |
|  | Min-Max | 9-582 | 7-227 |
|  | Missing | 1 (1.7%) | 1(1.9%) |
| NTproBNP (log scale) | Mean/SD | 7.42/1.23 | 7.57/1.36 |
|  | Median (Q1-Q3) | 7.43(6.50- 8.20) | 7.43(6.68- 8.74) |
| NTproBNP (original scale) | Mean/SD | 3603.17/6134.30 | 4435.79/6259.45 |
|  | Median (Q1-Q3) | 1683(665, 3686) | 1685.5(797, 6234) |
|  | Min-Max | 95-32594 | 89-31706 |
|  | Missing | 1 (1.7%) | 1(1.9%) |
| Urine albumin creatinine ratio (log scale) | Mean/SD | 1.65/1.21 | 1.83/1.37 |
|  | Median (Q1-Q3) | 1.21(0.59- 2.54) | 1.48(0.99- 2.81) |
| Urine albumin creatinine ratio (original scale) | Mean/SD | 29.53/87.54 | 21.00/61.15 |
|  | Median (Q1-Q3) | 3.35(1.8, 12.9) | 4.4(2.7, 16.6) |
|  | Min-Max | 0.4-514.3 | 0.8-388.7 |
|  | Missing | 11(18.6%) | 12(22.6%) |
| GUIDELINE-BASED MEDICAL THERAPY PRIOR TO RANDOMISATION | | | |
| Betablockers | yes | 53 (89.8%) | 51 (96.2%) |
| ACEi/ARB/ARNI | yes | 48 (81.4%) | 41 (77.4%) |
| MRA | yes | 27 (45.8%) | 20 (37.7%) |
| SGLT2i | yes | 37 (62.7%) | 38 (71.7%) |

## Supplementary table S2

**Table S2:** Sensitivity analyses for the trial's primary composite endpoint

Legend: Sensitivity analyses for the trial's primary composite endpoint—achieving the target RAASi dose while maintaining serum potassium below 5.6 mmol/L—under four extreme scenarios for missing observations. PP (N=96) results represent inference from logistic regression; ITT (N=112) results represent inference from multiple imputation (MI, 50 imputations) under the missing-at-random (MAR) assumption. OR = odds ratio, estimated for each scenario with p-values and 95% confidence intervals (CI).

| **SENSITIVITY ANALYSES SCENARIOS** | **OR: SZC vs. PLACEBO** | **p-value** | **95%CI low** | **95%CI high** |
| --- | --- | --- | --- | --- |
| PP ANALYSIS (COMPLETE DATA ANALYSIS) **(DF==1)** | 3.200 | 0.007 | 1.370 | 7.473 |
| ALL MISSING OBSERVATIONS SET TO 0 **(DF==1)** | 2.428 | 0.029 | 1.094 | 5.389 |
| ALL MISSING OBSERVATIONS SET TO 1 **(DF==1)** | 3.238 | 0.003 | 1.493 | 7.022 |
| MISSING OBSERVATIONS: PLACEBO=0, SZC=1 **(DF==1)** | 5.249 | <0.001 | 2.332 | 11.818 |
| MISSING OBSERVATIONS: PLACEBO=1, SZC=0 **(DF==1)** | 1.498 | 0.297 | 0.701 | 3.200 |
| **ITT ANALYSIS UNDER MAR (OBSERVED DATA ANALYSIS) (DF==NA)** | 3.159 | 0.008 | 1.350 | 7.392 |

## Supplementary table S3

**Table S3:** Potassium levels at each visit defined four longitudinal outcomes: hyperkalaemia, severe hyperkalaemia, and observed continuous values and their changes from one visit from the previous. Generalized estimating equations (GEE) derived population-average probabilities of (severe) hyperkalaemia across the two groups, while mixed (random effects) models provided subject-specific inference. The two methods - whilst yielding similar results for continuous outcomes irrespective of the approach - differ in parameter estimation methodology for binary outcomes (hyperkalaemia), interpretation, and missing-data assumptions—MCAR for GEE, MAR for binary random-effects models. Analyses of potassium changes were adjusted for baseline potassium values, including baseline (first visit) values in the continuous longitudinal models. Covariates include time and intervention group; no interaction between the two was observed in any model.

| **OUTCOMES** | **MODELS’ COVARIATES** | **OR** | **p-value** | **95%CI-low** | **95%CI-high** |
| --- | --- | --- | --- | --- | --- |
|  |  |  |  |  |  |
| **Potassium >=5.6mmol/L (Yes/No)** |  |  |  |  |  |
| GEE | Time(the effect of 2 weeks) | 1.236 | 0.001 | 1.088 | 1.405 |
| (Population average estimates) | Sodium Zirconium vs. Placebo | 0.379 | <0.001 | 0.225 | 0.639 |
| Random effects logit | Time(the effect of 2 weeks) | 1.286 | 0.001 | 1.109 | 1.492 |
| (Subject specific estimates) | Sodium Zirconium vs. Placebo | 0.342 | 0.009 | 0.153 | 0.763 |
| **Potassium >=6mmol/L (Yes/No)** |  |  |  |  |  |
| GEE | Time(the effect of 2 weeks) | 1.432 | 0.005 | 1.114 | 1.841 |
| (Population average estimates) | Sodium Zirconium vs. Placebo | 0.291 | 0.031 | 0.095 | 0.892 |
| Random effects logit | Time(the effect of 2 weeks) | 1.492 | 0.005 | 1.131 | 1.970 |
| (Subject specific estimates) | Sodium Zirconium vs. Placebo | 0.269 | 0.045 | 0.074 | 0.970 |
|  |  | **ESTIMATE** | **p-value** | **95%CI-low** | **95%CI-high** |
| **Potassium values as** | Time (the effect of 2 weeks) | 0.024 | 0.035 | 0.002 | 0.047 |
| **longitudinal continuous variable** | Sodium Zirconium vs. Placebo | -0.316 | <0.001 | -0.397 | -0.234 |
|  | Placebo group – avg. baseline | 5.021 |  | 4.949 | 5.094 |
|  | Sodium Zirconium– avg. baseline | 4.706 |  | 4.631 | 4.781 |
| **Potassium changes as** | Time (the effect of 2 weeks) | 0.030 | 0.049 | 0.001 | 0.060 |
| **longitudinal continuous variable** | Sodium Zirconium vs. Placebo | -0.079 | 0.134 | -0.182 | 0.024 |
|  | Potassium baseline | -0.073 |  | -0.205 | 0.059 |

## Supplementary table S4

**Table S4:** Mixed-modelling inference for laboratory outcomes

Legend: Mixed-modelling inference for laboratory outcomes, measured longitudinally at each visit. eGFR declined slowly but significantly over time in both groups (p<0.001), with no evidence of an intervention effect. No other measurements showed evidence of change over time within groups or differences between groups. Missing observations are assumed missing at random. The estimated mean baseline values for each measurement, by intervention group, are also presented.

| **BIOCHEMICAL MARKER** |  | **ESTIMATE** | **p-value** | **95%CI-low** | **95%CI-high** | **ESTIMATE** | **p-**  **value** | **95%CI-low** | **95%CI-high** |
| --- | --- | --- | --- | --- | --- | --- | --- | --- | --- |
|  |  | **OBSERVED LONGITUDINAL MEASUREMENTS** | | | | **CHANGES (CURRENT-PREVIOUS) WITH TIME** | | | |
| **eGFR** | Time (the effect of 2 weeks) | -0.920 | <0.001 | -1.406 | -0.433 | -0.227 | 0.104 | -0.499 | 0.046 |
|  | Sodium Zirconium vs. Placebo | -0.999 | 0.267 | -2.765 | 0.766 | 0.153 | 0.748 | -0.780 | 1.086 |
|  | Placebo group – avg. baseline | 36.557 |  | 34.987 | 38.127 |  |  |  |  |
|  | Sodium Zirconium– avg. baseline | 35.558 |  | 33.940 | 37.175 |  |  |  |  |
|  | Baseline effect (change only) |  |  |  |  | -0.051 | 0.014 | -0.092 | -0.010 |
| **Sodium levels** | Time (the effect of 2 weeks) | -0.115 | 0.108 | -0.255 | 0.025 | 0.055 | 0.445 | -0.086 | 0.195 |
|  | Sodium Zirconium vs. Placebo | 0.353 | 0.175 | -0.156 | 0.862 | 0.232 | 0.342 | -0.247 | 0.712 |
|  | Placebo group – avg. baseline | 138.676 |  | 138.224 | 139.129 |  |  |  |  |
|  | Sodium Zirconium– avg. baseline | 139.029 |  | 138.563 | 139.496 |  |  |  |  |
|  | Baseline effect (change only) |  |  |  |  | -0.056 | 0.181 | -0.138 | 0.026 |
| **Troponin levels** | Time (the effect of 2 weeks) | 0.014 | 0.371 | -0.017 | 0.044 | 0.001 | 0.847 | -0.014 | 0.012 |
| **(continuous -log scale)** | Sodium Zirconium vs. Placebo | 0.015 | 0.791 | -0.096 | 0.126 | 0.001 | 0.973 | -0.044 | 0.046 |
|  | Placebo group – avg. baseline | 3.450 |  | 3.351 | 3.549 |  |  |  |  |
|  | Sodium Zirconium– avg. baseline | 3.465 |  | 3.364 | 3.566 |  |  |  |  |
|  | Baseline effect (change only) |  |  |  |  | -0.008 | 0.619 | -0.042 | 0.025 |
| **NTproBNPC levels** | Time (the effect of 2 weeks) | 0.028 | 0.331 | -0.028 | 0.082 | 0.010 | 0.398 | -0.013 | 0.033 |
| **(continuous -log scale)** | Sodium Zirconium vs. Placebo | 0.116 | 0.256 | -0.084 | 0.316 | 0.021 | 0.609 | -0.058 | 0.100 |
|  | Placebo group – avg. baseline | 7.308 |  | 7.130 | 7.486 |  |  |  |  |
|  | Sodium Zirconium– avg. baseline | 7.424 |  | 7.241 | 7.608 |  |  |  |  |
|  | Baseline effect (change only) |  |  |  |  | -0.008 | 0.619 | -0.041 | 0.024 |
| **ACR levels** | Within groups effect | -0.003 | 0.515 | -0.010 | 0.005 | 0.010 | 0.207 | -0.006 | 0.026 |
| **(continuous -log scale)** | Sodium Zirconium vs. Placebo | 0.317 | 0.221 | -0.190 | 0.824 | 0.255 | 0.293 | -0.226 | 0.736 |
| **Only 2 measurements** | Placebo group – avg. baseline | 1.513 |  | 1.115 | 1.910 |  |  |  |  |
|  | Sodium Zirconium– avg. baseline | 1.830 |  | 1.409 | 2.251 |  |  |  |  |
|  | Baseline effect (change only) |  |  |  |  | -0.085 | 0.293 | -0.245 | 0.075 |

## Supplementary table S5

**Table S**5: Specific agents used during the study in all patients

| **Agent** | **At baseline** | **At study end** |
| --- | --- | --- |
| **RASi (ACEi/ARB/ARNI)** | | |
| Sacubitril/Valsartan | 83 | 82 |
| Ramipril | 16 | 13 |
| Candesartan | 8 | 7 |
| Losartan | 1 | 1 |
| Irbesartan |  | 1 |
| **MRA** | | |
| Eplerenone | 37 | 58 |
| Spironolactone | 19 | 27 |

Legend: Table shows the specific RASi and MRA agents used within the trial at the start of study and study end. *RASi – renin-angiotensin system inhibitor; MRA – mineralocorticoid receptor antagonist*

## Supplementary table S6

**Table S6:** Dose escalations across groups

| **Medication escalations** | **Rate Ratio** | **p-value** | **95%CI LOW** | **95%CI HIGH** |
| --- | --- | --- | --- | --- |
| (number per two weeks) | Sodium Zirconium vs. Placebo |  |  |  |
| RASi escalations | 1.127 | 0.408 | 0.849 | 1.497 |
| MRA escalations | 1.015 | 0.935 | 0.717 | 1.436 |
| Total escalations | 1.074 | 0.451 | 0.892 | 1.293 |

Legend: There is no evidence to suggest that the rate of escalation in RASi, MRA and both were different between the two groups.
*CI – confidence interval; RASi – renin-angiotensin system inhibitor; MRA – mineralocorticoid receptor antagonist*

The number of RAAS inhibition medication escalations per participant was modelled as a Poisson count, with the total time spent in the trial included as an offset to investigate whether the rates of escalation differed by intervention groups during the follow-up. The analyses inferred the rate ratios of escalations between the Sodium Zirconium and Placebo groups. The results are consistent with no difference between groups, irrespective of medication escalation, RASi, MRA or total.

## Supplementary table S7

**Table S7:** Evaluation of patient attrition by withdrawal motives.

***Missing data analysis evaluation:***

Legend: A total of 6 out of 59 patients (10.2%) in the Placebo group and 10 out of 53 (18.9%) in the Sodium Zirconium group withdrew from the study (p = 0.189, chi-squared test, DF=1). A more detailed breakdown of withdrawal reasons, as shown below, also revealed no significant difference between groups (p = 0.209, Fisher’s exact test, DF not applicable). Two-sided p-values have been inferred.

|  | **SODIUM ZIRCONIUM** | **PLACEBO** | **TOTAL** |
| --- | --- | --- | --- |
| Finished the trial | 43(81%) | 53(90%) | 96(86%) |
| Clinical decision | 2(4%) | 3(5%) | 5(4%) |
| Patient decision | 8(15%) | 3(5%) | 11 (10%) |

## Supplementary table S8

**Table S9:** Evaluation of patient attrition by physical factors.

Legend: Some evidence suggests that the attrition is inversely associated with sodium levels, namely the higher levels of the sodium levels the less likely is the participant’s attrition but there was no difference between the two groups in Sodium levels at the baseline as expected. There is no evidence to suggest any other patterns in the participants attrition by group, by other baseline characteristics or characteristics adjusted for in the intervention or the interaction between them. A binary variable has been defined indicating whether a patient was lost at the follow-up or not and a logistic regression has been conducted. Two-sided p-values have been inferred. This is an exploratory analysis and no multiple testing adjustment has been considered.

| **Variables** | **OR** | **p-value** | **95%CI LOW** | **95%CI HIGH** |
| --- | --- | --- | --- | --- |
| **Sodium Zirconium vs. Placebo** | 2. 054 | 0.195 | 0.691 | 6.105 |
| Age at recruitment (the effect of 1 year) | 0.988 | 0.606 | 0.943 | 1.035 |
| Male vs Female | 0.556 | 0.300 | 0.183 | 1.690 |
| Non-White vs. White | 2.310 | 0.126 | 0.791 | 6.752 |
| Diabetes: Yes vs. No | 0.605 | 0.356 | 0.208 | 1.758 |
| Hypertension: Yes vs. No | 0.467 | 0.170 | 0.157 | 1.387 |
| IHD: Yes vs. No | 1.403 | 0.558 | 0.451 | 4.365 |
| Hypercholesterolaemia: Yes vs. No | 0.464 | 0.168 | 0.156 | 1.382 |
| Smoking: Ever vs. Never | 1.380 | 0.578 | 0.444 | 4.289 |
| Potassium | 1.480 | 0.586 | 0.361 | 6.069 |
| Sodium | 0.765 | **0.002** | 0.643 | 0.910 |
| eGFR | 1.010 | 0.673 | 0.965 | 1.056 |
| Troponin (log scale) | 1.180 | 0.651 | 0.576 | 2.417 |
| BNP (log scale) | 1.137 | 0.543 | 0.752 | 1.719 |
| ACR (log scale) | 0.995 | 0.982 | 0.642 | 1.542 |

## Supplementary table S9

**Table S9:** The cause of admission during follow up

Legend: Admission causes for severe adverse events within trial stratified by treatment arm. *ICD – implantable cardiac defibrillator; NSTEMI – non-ST elevation myocardial infarction; UTI – urinary tract infection*

| **Admission Causes** | **Intervention** | **Placebo** | **Grand Total** |
| --- | --- | --- | --- |
| Arrhythmia |  | 3 | 3 |
| Blood Pressure |  | 1 | 1 |
| Elective Insertion of ICD |  | 1 | 1 |
| Electrolytes |  | 2 | 2 |
| Falls |  | 1 | 1 |
| Heart Failure, decompensated | 1 | 3 | 4 |
| ICD problems |  | 1 | 1 |
| UTI, with renal calculus |  | 1 | 1 |
| NSTEMI | 1 |  | 1 |
| **Grand Total** | 2 | 13 | 15 |

## Supplementary table S10

Table S10: Instructions for changing the dose of IMP at every 2-weekly study visits

Legend: The IMP (SZC or placebo) was changed depending on serum potassium and previous dose of IMP. IMP – investigational medicinal product; SZC – sodium zirconium cyclosilicate

| **SZC or Placebo dose at start of visit** | **Serum potassium end of visit** | **Dose change at the end of visit** |
| --- | --- | --- |
| If on 10 g daily | > 5.0 mmol/L  (but ≤ 6.0 mmol/L) | Continue 10 g daily |
|  | 3.5–5.0 mmol/L | Change to 5 g daily |
|  | < 3.5 mmol/L | Temporary stop |
|  | > 6.0 mmol/L | Stop and initiate rescue therapy |
| If on 5 g daily | > 5.0 mmol/L | Change to 10 g daily |
|  | 3.5–5.0 mmol/L | Continue 5 g daily |
|  | < 3.5 mmol/L | Temporary stop |
| If on 0 g daily (temporary stop) | ≤ 5.0 mmol/L | Continue with no IMP |
|  | > 5.0 mol/L | Initiate 5 g daily |

## Supplementary table S11

Table S11: The minimum, half-maximal, and maximum dose of ACEi/ARB/ARNI and MRA

|  | **Minimum dose** | **Half-maximal dose** | **Maximum dose** |
| --- | --- | --- | --- |
| **ACEi/ARB/ARNI**  **(one of the following)** | | | |
| Ramipril | 2.5 mg OD | 5mg OD | 10mg OD |
| Perindopril arginine | 2.5 mg OD | 5mg OD | 10mg OD |
| Enalapril | 2.5 mg OD | 5mg OD | 10 / 20 / 40mg OD |
| Lisinopril | 2.5 mg OD | 5 / 10mg OD | 20 / 40mg OD |
| Perindopril erbumine | 2 mg OD | 4mg OD | 8 / 16mg OD |
| Sacubitril/ valsartan | 24 / 26 mg BD | 49 / 51mg BD | 97 / 103mg BD |
| Losartan | 25 mg OD | 50mg OD | 100mg OD |
| Candesartan | 4 mg OD | 8 / 16mg OD | 32mg OD |
| Irbesartan | 75 mg OD | 150mg OD | 300 mg OD |
| Telmisartan | 20mg OD | 40mg OD | 80mg OD |
| Olmesartan | 10mg OD | 20mg OD | 40mg OD |
| Valsartan | 40mg BD | 80mg BD | 160 mg BD |
| **MRA (one of the following)** | | | |
| Spironolactone | 12.5mg OD | 25mg OD | 50 g OD |
| Eplerenone | 12.5mg OD | 25mg OD | 50mg OD |

Legend: The study clinician was instructed to increase from starting, to half-maximum, and maximum dose provided it was safe to do so. *BD – twice a day; OD – once a day; ARNI – Angiotensin receptor neprilysin inhibitor; MRA – mineralocorticoid receptor antagonist*

## Supplementary table S12

Table S12: Guidance on dose escalation of RASi(ACEi/ARB/ARNI) and MRA in 2-weekly study visits

|  | **Ideal Doses** | | | | | | |
| --- | --- | --- | --- | --- | --- | --- | --- |
| **Visits** | **Visit 1** | **Visit 2** | **Visit 3** | **Visit 4** | **Visit 5** | **Visit 6** | **Visit 7** |
| **RASi (ACEi/ARB/ARNI)** **(one of the following)** | | | | | | | |
| Sacubitril/Valsartan  *(Twice a day)* | 0mg | 24/26mg | 24/26 mg | 49/51 mg | 49/51 mg | 97/103 mg | 97/103 mg |
| Ramipril  *(once a day)* | 0mg | 2.5mg | 2.5 mg | 5mg | 5mg | 10 mg | 10 mg |
| Candesartan  *(once a day)* | 0mg | 4 mg | 4 mg | 8 mg | 16 mg | 32 mg | 32 mg |
| *Dose Categorisation* | None | Minimum dose | | Half maximal dose | | Maximum dose | |
| **MRA (one of the following)** | | | | | | | |
| Eplerenone  (Once a day) | 0mg | 0mg | 12.5mg | 12.5mg | 25mg | 25mg | 50 mg |
| Spironolactone  (Once a day) | 0mg | 0mg | 12.5mg | 12.5mg | 25 mg | 25 mg | 50 mg |
| *Dose Categorisation* | None | | Minimum dose | | Half maximal dose | | Maximum dose |

Legend: Table showing how dose could have been escalated in a patient starting with no RAASi where maximum dose can be achieved over 7 study visits. *RASi – renin-angiotensin system inhibitor; MRA – mineralocorticoid receptor antagonist*

## Supplementary Figure S1 A and B

Figure S1 RAASi dose in each visit during the study

A

B

Legend: The observed dose of ACEi/ARB/ARNI (A); The observed dose of MRA in 2-weekly study visits (B) : The bars indicate the number of participants on none, minimum dose, half maximum, and maximum dose of RASi and MRA in visits 1-7 two weeks apart stratified by SZC and Placebo arms. *RASi – renin-angiotensin system inhibitor; MRA – mineralcorticoid receptor antagonist; SZC – sodium zirconium cyclosilicate*

## Supplementary note 1

**CONSORT 2025 checklist of information to include when reporting a randomised trial***

| **Section / Topic** | **No** | **CONSORT 2025 checklist item description** | **Reported on page no.** |
| --- | --- | --- | --- |
| **Title and abstract** | | |  |
| Title and structured abstract | 1a | Identification as a randomised trial | Title |
|  | 1b | Structured summary of the trial design, methods, results, and conclusions | Abstract, p. 2 |
| **Open science** | | |  |
| Trial registration | 2 | Name of trial registry, identifying number (with URL) and date of registration | p. 2 |
| Protocol and statistical analysis plan | 3 | Where the trial protocol and statistical analysis plan can be accessed | p. 16,  Supplementary |
| Data sharing | 4 | Where and how the individual de-identified participant data (including data dictionary), statistical code and any other materials can be accessed | p. 21 |
| Funding and conflicts of interest | 5a | Sources of funding and other support (e.g., supply of drugs), and role of funders in the design, conduct, analysis and reporting of the trial | p. 20, p. 24 |
|  | 5b | Financial and other conflicts of interest of the manuscript authors | p. 24 |
| **Introduction** | | |  |
| Background and rationale | 6 | Scientific background and rationale | p. 3 |
| Objectives | 7 | Specific objectives related to benefits and harms | p. 3 |
| **Methods** | | |  |
| Patient and public involvement | 8 | Details of patient or public involvement in the design, conduct and reporting of the trial | Supplementary - Protocol |
| Trial design | 9 | Description of trial design including type of trial (e.g., parallel group, crossover), allocation ratio, and framework (e.g., superiority, equivalence, non-inferiority, exploratory) | p. 13,  Supplementary - Protocol |
| Changes to trial protocol | 10 | Important changes to the trial after it commenced including any outcomes or analyses that were not prespecified, with reason | p. 14,  Supplementary - Protocol |
| Trial setting | 11 | Settings (e.g., community, hospital) and locations (e.g., countries, sites) where the trial was conducted | p. 13,  Supplementary - Protocol |
| Eligibility criteria | 12a | Eligibility criteria for participants | p. 13-14,  Supplementary - Protocol |
|  | 12b | If applicable, eligibility criteria for sites and for individuals delivering the interventions (e.g., surgeons, physiotherapists) | Supplementary - Protocol |
| Intervention and comparator | 13 | Intervention and comparator with sufficient details to allow replication. If relevant, where additional materials describing the intervention and comparator (e.g., intervention manual) can be accessed | p. 14-15 Supplementary - Protocol |
| Outcomes | 14 | Pre-specified primary and secondary outcomes, including the specific measurement variable (e.g., systolic blood pressure), analysis metric (e.g., change from baseline, final value, time to event), method of aggregation (e.g., median, proportion), and time point for each outcome | p. 15-16 |
| Harms | 15 | How harms were defined and assessed (e.g., systematically, non-systematically) | p. 21-22 |
| Sample size | 16a | How sample size was determined, including all assumptions supporting the sample size calculation | p. 17 |
|  | 16b | Explanation of any interim analyses and stopping guidelines | p. 21 |
| Randomisation: |  |  |  |
| Sequence generation | 17a | Who generated the random allocation sequence and the method used | p. 16 |
|  | 17b | Type of randomisation and details of any restriction (e.g., stratification, blocking and block size) | p. 16 |
| Allocation concealment mechanism | 18 | Mechanism used to implement the random allocation sequence (e.g., central computer/telephone; sequentially numbered, opaque, sealed containers), describing any steps to conceal the sequence until interventions were assigned | p. 16 |
| Implementation | 19 | Whether the personnel who enrolled and those who assigned participants to the interventions had access to the random allocation sequence | p. 16 |
| Blinding | 20a | Who was blinded after assignment to interventions (e.g., participants, care providers, outcome assessors, data analysts) | p. 16 |
|  | 20b | If blinded, how blinding was achieved and description of the similarity of interventions | p. 16 |
| Statistical methods | 21a | Statistical methods used to compare groups for primary and secondary outcomes, including harms | p. 17-20 |
|  | 21b | Definition of who is included in each analysis (e.g., all randomised participants), and in which group | p. 5 |
|  | 21c | How missing data were handled in the analysis | p. 20 |
|  | 21d | Methods for any additional analyses (e.g., subgroup and sensitivity analyses), distinguishing prespecified from post-hoc | p. 8-9 |
| **Results** | | |  |
| Participant flow, including flow diagram | 22a | For each group, the numbers of participants who were randomly assigned, received intended intervention, and were analysed for the primary outcome | p.5,  Figure 1 |
|  | 22b | For each group, losses and exclusions after randomisation, together with reasons | p.5,  Figure 1 |
| Recruitment | 23a | Dates defining the periods of recruitment and follow-up for outcomes of benefits and harms | p. 13-14 |
|  | 23b | If relevant, why the trial ended or was stopped | Not relevant |
| Intervention and comparator delivery | 24a | Intervention and comparator as they were actually administered (e.g., where appropriate, who delivered the intervention/comparator, how participants adhered, whether they were delivered as intended [fidelity]) | p. 14-15 |
|  | 24b | Concomitant care received during the trial for each group | p. 14-15 |
| Baseline data | 25 | A table showing baseline demographic and clinical characteristics for each group | Table 1 |
| Numbers analysed,  outcomes and estimation | 26 | For each primary and secondary outcome, by group:   - the number of participants included in the analysis - the number of participants with available data at the outcome time point - result for each group, and the estimated effect size and its precision (such as 95% confidence interval) - for binary outcomes, presentation of both absolute and relative effect size | p. 5-9 |
| Harms | 27 | All harms or unintended events in each group | p. 8 |
| Ancillary analyses | 28 | Any other analyses performed, including subgroup and sensitivity analyses, distinguishing pre-specified from post-hoc | p. 8-9 |
| **Discussion** | | |  |
| Interpretation | 29 | Interpretation consistent with results, balancing benefits and harms, and considering other relevant evidence | p. 8-12 |
| Limitations | 30 | Trial limitations, addressing sources of potential bias, imprecision, generalisability, and, if relevant, multiplicity of analyses | p. 12 |

*We strongly recommend reading this statement in conjunction with the CONSORT 2025 Explanation and Elaboration and/or the CONSORT 2025 Expanded Checklist for important clarifications on all the items. We also recommend reading relevant CONSORT extensions. See [www.consort-spirit.org](http://www.consort-spirit.org).

Citation: Hopewell S, Chan AW, Collins GS, Hróbjartsson A, Moher D, Schulz KF, et al. CONSORT 2025 Statement: updated guideline for reporting randomised trials. BMJ. 2025; 388:e081123. <https://dx.doi.org/10.1136/bmj-2024-081123>.

© 2025 Hopewell et al. This is an Open Access article distributed under the terms of the Creative Commons Attribution License (<https://creativecommons.org/licenses/by/4.0/>), which permits unrestricted use, distribution, and reproduction in any medium, provided the original work is properly cited.
